# Health Benefits and Food Applications of Bioactive Constituents From Different Medicinal Parts (Bark, Leaf, and Fruit) of *Lycium barbarum*

**DOI:** 10.1002/fsn3.71018

**Published:** 2025-09-30

**Authors:** Mengfan Peng, Caixia Li, Xue Yang, Tingting Ye, Baosong Liu

**Affiliations:** ^1^ Faculty of Medicine HuangHuai University Zhumadian China; ^2^ NMPA Key Laboratory for Safety Research and Evaluation of Traditional Chinese Medicine Henan University of Chinese Medicine Zhengzhou China; ^3^ Department of Pharmacy Zhumadian Traditional Chinese Medicine Hospital Zhumadian China; ^4^ The First Affiliated Hospital of Xinxiang Medical University Xinxiang China

**Keywords:** bioactive ingredients, food products, *Lycium barbarum*, pharmacological effects

## Abstract

*Lycium barbarum* is a plant that is a member of the Solanaceae family. Different tissues and organs from this plant are widely used in traditional Chinese medicine because of their abundant functional ingredients. Recently, *Lycium barbarum* has been the subject of several ethnopharmacological studies. With the deepening research and development of products utilizing functional ingredients, the edible value of *Lycium barbarum* has been gradually explored, attracting the attention of scholars due to its excellent nutritional properties. In 1988, the fruits of *Lycium barbarum* were officially included in the list of “both medicine and food” by the Chinese Ministry of Health. Additionally, the bark and leaves of *Lycium barbarum* are edible. Owing to its dual‐use characteristics in medicine and food, *Lycium barbarum* has been widely used as a traditional Chinese medicine and functional food worldwide. However, a comprehensive summary focused on the functional ingredients, pharmacological effects, food research and development status, and safety of *Lycium barbarum* is lacking. Therefore, we reviewed the major functional ingredient categories, structural characteristics, and nutritional value. Furthermore, we summarize the pharmacological effects of the functional ingredients to support their further application as therapeutic agents. Food research and the development of functional ingredients from *Lycium barbarum* are also systematically reviewed. Finally, this review provides a comprehensive overview of the safety and future development of products derived from *Lycium barbarum* , exploring potential applications in the fields of cosmetics, agriculture, functional foods, and nutritional supplements of its bioactive constituents, with the aim of facilitating further extensive investigations into its utilization.

## Graphical abstract

The bioactive ingredients, pharmacological effects, food applications, and safety of *Lycium barbarum*.

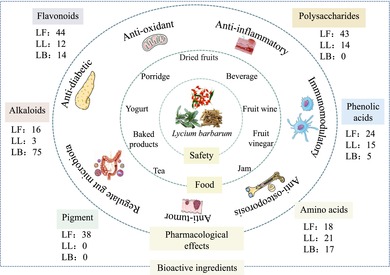

## 1 Introduction

**FIGURE 1:**
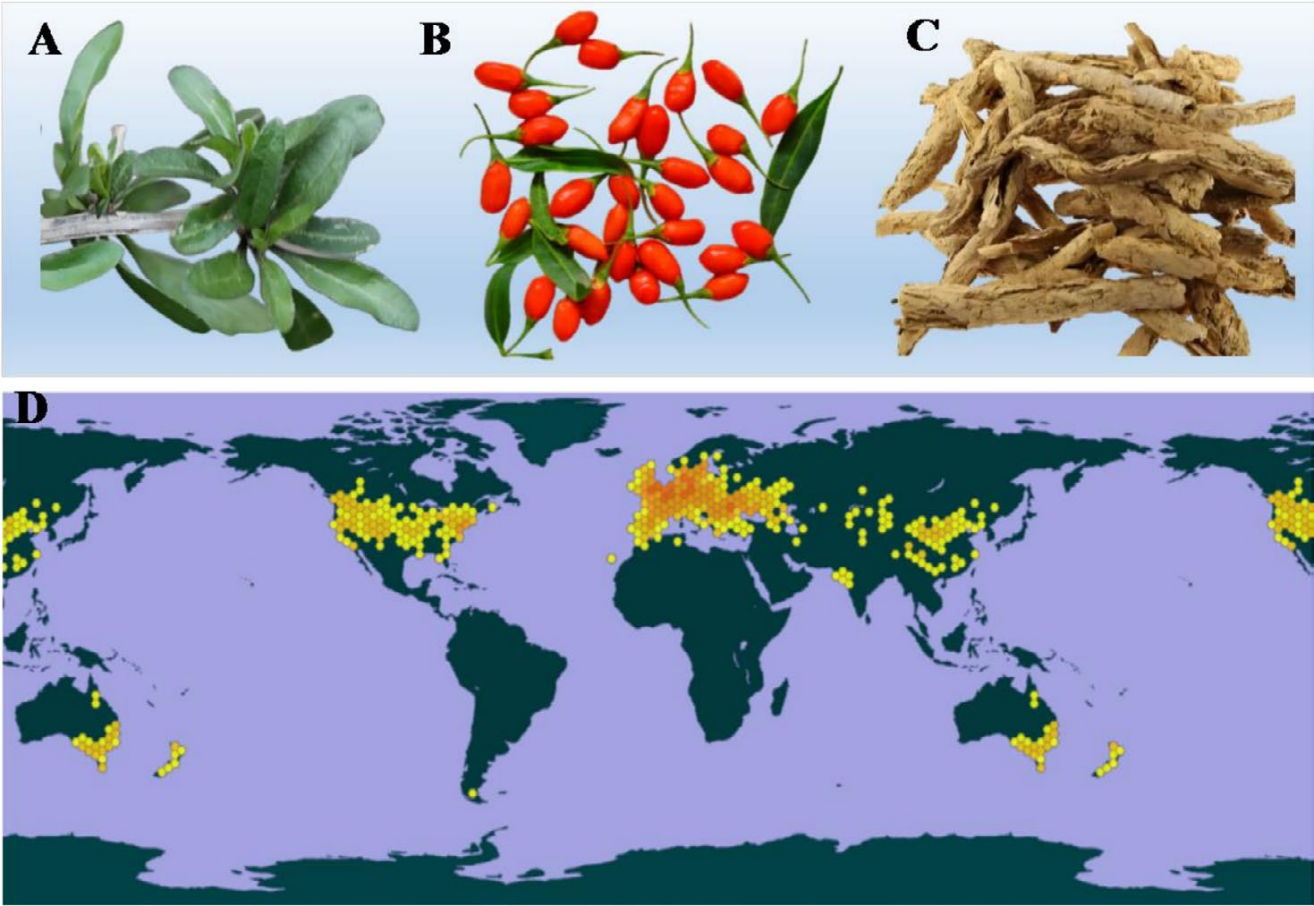
(A) The leaves of *Lycium barbarum* . (B) The fruits of *Lycium barbarum* . (C) The dried root bark of *Lycium barbarum* . (D) The distribution diagram of *Lycium barbarum* in the world.

*Lycium barbarum* is a plant of the Solanaceae family and Lycium L. genus that is native to temperate regions of East Asia and is primarily distributed in arid and semiarid areas of China, South Korea, Europe, Japan, and the Mediterranean (Figure 1). China is the major supplier of *Lycium barbarum* products worldwide, with *Lycium barbarum* widely distributed in the northwest region of China, including Xinjiang, Qinghai, Gansu, Inner Mongolia, and Ningxia (Zeng 2022). The medicinal value of *Lycium barbarum* was first described in “Shennong Bencao Jing,” which states that “long‐term consumption strengthens muscles and bones, lightens the body, and prevents aging.” *Lycium barbarum* is a veritable treasure trove of leaves (LL), bark (LB), and fruits (LF), all of which have been utilized throughout the history of traditional medicine. Flavonoids, polysaccharides, alkaloids, phenolic acids, *Lycium barbarum* pigments, and amino acids are the major bioactive ingredients of different *Lycium barbarum* tissues, and an increasing number of these compounds have been successfully extracted from various parts of *Lycium barbarum* with continuous improvements in extraction and analytical technology (Zhou et al. 2022). As the details of the structure and properties of these diverse components become increasingly clear, *Lycium barbarum* has been demonstrated to exhibit notable biological activities and therapeutic effects, including antioxidant, antidiabetic, anti‐inflammatory, antiosteoporosis, immune regulation, and anticancer effects, as well as regulation of the gut microbiota (Zhang, Dai, et al. 2024). LL, LB, and LF are rich in nutrients, such as protein, vitamin C, trace elements, and amino acids, and they also possess both edible and medicinal value. They have been developed into tea drinks, alcoholic beverages, and health foods, and are popular both domestically and internationally. Additionally, LL has been used to cook soups, stir‐fried eggs, and boiled eggs in many Asian countries. However, to date, the nutritional health and comprehensive utilization of *Lycium barbarum* have not been thoroughly reviewed in the fields of food and health (Duan et al. 2024). Therefore, we comprehensively summarized the bioactive ingredients, pharmacological effects, safety, food applications, and improvements in *Lycium barbarum* to highlight the health benefits of this plant.

## 2 Types of Bioactive Ingredients of *Lycium barbarum*

**FIGURE 2:**
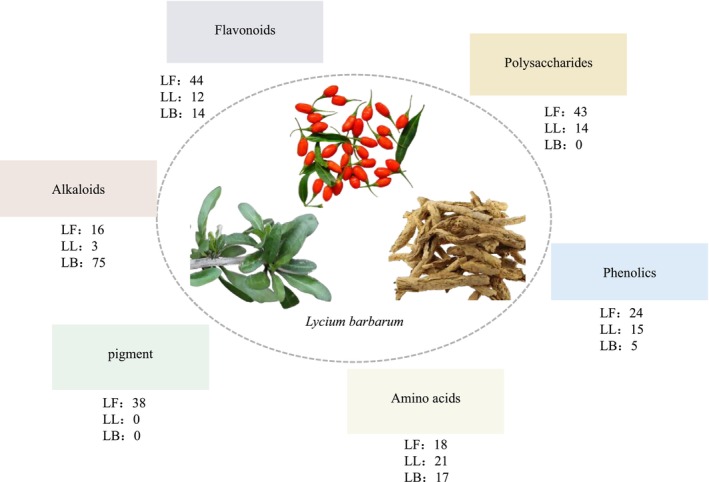
Distribution overview of the main chemical components of *Lycium barbarum* in LF, LL, and LB.

To date, at least 285 compounds, including flavonoids, polysaccharides, alkaloids, phenolic acids, *Lycium barbarum* pigments, and amino acids, have been isolated and identified from *Lycium barbarum* . However, differences have been observed in the types and quantities of compounds in the different parts of the plant. Among these, LF possesses the greatest number of flavonoids, polysaccharides, and phenolic compounds, with counts of 44, 43, and 24, respectively. Notably, 38 *Lycium barbarum* pigments are present in LF; however, no relevant reports have been identified for LL and LB. Alkaloids are the major components of LBs (up to 75 compounds), followed by flavonoids. In LL, amino acid compounds are the most abundant, followed by phenolic acids, polysaccharides, and flavonoids. Although the major categories of the components in LF, LL, and LB are essentially consistent and all contain flavonoids, polysaccharides, alkaloids, phenols, amino acids, and other components, there are certain differences. These differential components can serve as corresponding quality markers to distinguish the three from each other. For example, *Lycium barbarum* pigments and polysaccharides (54–72, 78–97, 107–110) can be used as markers for LF to distinguish it from LL and LB; alkaloids (111–123, 125–126, 128–129, 131–176, 180–181, and 191–192) can serve as markers for LB to differentiate it from LF and LL; and 4‐aminobutyric acid, citrulline, and ornithine, as well as 73–77 and 98–106 among polysaccharides, can act as markers for LL to distinguish it from LF and LB (Table 1, Figure 2).

**TABLE 1:** Bioactive ingredients isolated from the barks, leaves, male flowers, and seeds of *Lycium barbarum* .

No.	Classification/Compound name	Source	References
**Flavonoids**			
1	Naringenin	LF; LL	Zhang, Dai, et al. (2024); (Ma 2024b)
2	Quercetin	LF; LL; LB	Zeng (2022); Yan et al. (2024)
3	Luteolin	LF; LL; LB	Zeng (2022); Zhao (2020); Lu et al. (2021)
4	Morin hydrate	LL	Ma (2024b)
5	Naringenin‐7‐*O*‐rutinoside	LF	Zhang, Dai, et al. (2024)
6	Naringenin‐7‐*O*‐glucoside	LF	Zhang, Dai, et al. (2024)
7	Isoquercetin	LF	Zhang, Dai, et al. (2024)
8	Rutin	LF; LL; LB	Zhang, Dai, et al. (2024); Zhao (2020); Yan et al. (2024)
9	Kaempferol	LF; LL; LB	Zhou et al. (2022); Ma (2024b); Lu et al. (2021)
10	Isorhamnetin	LF; LL	Zhang, Dai, et al. (2024); Ma (2024b)
11	Quercetin‐*O*‐rutinoside‐hexglycoside	LF	Zhang, Dai, et al. (2024)
12	Kaempferol‐3‐*O*‐rutinoside	LF, LL	Zhang, Dai, et al. (2024); Zhao (2020)
13	Isorhamnetin‐3‐*O*‐rutinoside	LF	Zhang, Dai, et al. (2024)
14	Dihydromyricetin‐*O*‐glucoside	LF	Zhang, Dai, et al. (2024)
15	Acacetin	LF; LL; LB	Zhou et al. (2022); Zhang, Dai, et al. (2024); Lu et al. (2021)
16	Catechin	LF; LL	Zhang, Dai, et al. (2024); Ma (2024b)
17	7‐*O*‐*β*‐D‐glucopyranosyl‐rutin	LF	Ma et al. (2024)
18	Narcissin	LF	Ma et al. (2024)
19	Apigenin	LB	Zhou et al. (2022)
20	Arbutin	LF	Zhou et al. (2022)
21	Buddleoside	LF; LB	Zhou et al. (2022); Lu et al. (2021)
22	Patuletin	LF	Zhou et al. (2022)
23	Myricetin	LF; LL	Zhou et al. (2022); Ma (2024b)
24	Hyperoside	LF	Ma et al. (2024)
25	5,7,3′‐trihydroxy‐6,4′, 5′‐trimethoxyflavone	LL	Zhou et al. (2022)
26	Pectolinarigenin‐7‐glucoside	LF	Ma (2024b)
27	Chrysoeriol‐7‐*O*‐glucoside	LF	Ma (2024b)
28	6‐hydroxy‐luteolin‐5‐glucoside	LF	Ma (2024b)
29	Quercetin‐5‐*O*‐*β*‐D‐glucoside	LF	Ma (2024b)
30	Hesperetin‐5‐*O*‐glucoside	LF	Ma (2024b)
31	Quercetin‐3‐*O*‐glucoside	LF	Ma (2024b)
32	Pinobanksin	LF	Ma (2024b)
33	Naringein chalcone	LF	Ma (2024b)
34	Isohyperoside	LF	Ma (2024b)
35	Isorhamnetin‐3‐*O*‐rhamnoside	LF	Ma (2024b)
36	Quercetin‐7‐*O*‐glucoside	LF	Ma (2024b)
37	Quercetin‐4′‐*O*‐glucoside	LF	Ma (2024b)
38	Quercetin‐3‐*O*‐sambubioside	LF	Ma (2024b)
39	Yuanhuanin	LF	Ma (2024b)
40	Gossypetinidin‐3‐*O*‐rutinoside	LF	Ma (2024b)
41	6‐ Methoxy kaempferol‐3‐*O*‐glucoside	LF	Ma (2024b)
42	Rhamnetin‐3‐*O*‐glucoside	LF	Ma (2024b)
43	Rhodioflavonoside	LF	Ma (2024b)
44	Iristectorigenin A	LF	Ma (2024b)
45	Nicotiflorin	LF	Ma et al. (2024)
46	Morin	LF	Ma et al. (2024)
47	Wogonin	LB	Wang et al. (2024)
48	Wogonin‐7‐*O*‐*β*‐D‐ethylglucuronide	LB	Wang et al. (2024)
49	Wogonin‐7‐*O*‐*β*‐D‐methylglucuronide	LB	Wang et al. (2024)
50	2′,5‐dihydroxy‐6,7,8,6′‐tetramethoxyflavone	LB	Lu et al. (2021)
51	Maackiain	LB	Lu et al. (2021)
52	Kazinol	LB	Lu et al. (2021)
53	Quercitrin	LF; LB	Zhang, Dai, et al. (2024), Lu et al. (2021)
**Polysaccharides**			
54	*Lycium ruthenicum* polysaccharide 4‐A (LRP4‐A)	LF	Zhang, Dai, et al. (2024)
55	*Lycium ruthenicum* glycoconjugate polysaccharide 1 (LRGP1)	LF	Zhang, Dai, et al. (2024)
56	*Lycium ruthenicum* glycoconjugate polysaccharide 1 (LRGP3)	LF	Zhang, Dai, et al. (2024)
57	*Lycium ruthenicum* glycoconjugate polysaccharide 5 (LRGP5)	LF	Zhang, Dai, et al. (2024)
58	(2S)‐2‐*O*‐linolenoyl‐3‐*O*‐[(3″‐*O*‐linolenoyl‐*α*‐D‐galactopyranose)‐(1″ → 6′)‐*β*‐D‐galactopyranose]‐1‐ *O*‐palmitoyl glycerol	LF	Zhang, Dai, et al. (2024)
59	(2S)‐2‐*O*‐linolenoyl‐3‐*O*‐[(3″‐*O*‐linoleoyl‐*α*‐D‐galactopyranose)‐(1″ → 6′)‐*β*‐D‐galactopyranose]‐1‐*O*‐palmitoyl glycerol	LF	Zhang, Dai, et al. (2024)
60	(2S)‐2‐*O*‐linolenoyl‐1‐*O*‐palmitoyl‐3‐*O*‐[(3″‐*O*‐palmitoyl‐*α*‐D‐galactopyranose)‐(1″ → 6′)‐*β*‐D‐ galactopyranose] glycerol	LF	Zhang, Dai, et al. (2024)
61	(2S)‐2‐*O*‐linoleoyl‐1‐*O*‐palmitoyl‐3‐*O*‐[(3″‐*O*‐palmitoyl‐*α*‐D‐galactopyranose)‐(1″ → 6′)‐*β*‐D‐ galactopyranose] glycerol	LF	Zhang, Dai, et al. (2024)
62	(2S)‐1,2‐*O*‐dipalmitoyl‐3‐*O*‐[(3″‐*O*‐palmitoyl‐*α*‐D‐galactopyranose)‐(1″ → 6′)‐*β*‐D‐galactopyranose] glycerol	LF	Zhang, Dai, et al. (2024)
63	(2S)‐1,2‐*O*‐dipalmitoyl‐3‐*O*‐[*α*‐D‐galactopyranose‐(1″ → 6′)‐*β*‐D‐galactopyranose] glycerol	LF	Zhang, Dai, et al. (2024)
64	(2S)‐3‐*O*‐[*α*‐D‐galactopyranose‐(1″ → 6′)‐*β*‐D‐galactopyranose]‐1‐*O*‐linolenoyl‐2‐*O*‐linoleoyl glycerol	LF	Zhang, Dai, et al. (2024)
65	(2S)‐3‐*O*‐[*α*‐D‐galactopyranose‐(1″ → 6′)‐*β*‐D‐galactopyranose]‐2‐*O*‐linolenoyl‐1‐*O*‐palmitoyl glycerol	LF	Zhang, Dai, et al. (2024)
66	(2S)‐3‐*O*‐[*α*‐D‐galactopyranose‐(1″ → 6′)‐*β*‐D‐galactopyranose]‐2‐*O*‐linoleoyl‐1‐*O*‐palmitoyl glycerol	LF	Zhang, Dai, et al. (2024)
67	(2S)‐3‐*O*‐[*α*‐D‐galactopyranose‐(1″ → 6′)‐*β*‐D‐galactopyranose]‐2‐*O*‐oleoyl‐1‐*O*‐palmitoyl glycerol	LF	Zhang, Dai, et al. (2024)
68	(2S)‐1‐*O*‐stearoyl‐2‐*O*‐linoleoyl‐3‐*O*‐[*α*‐D‐galactopyranose‐(1″ → 6′)‐*β*‐D‐galactopyranose] glycerol	LF	Zhang, Dai, et al. (2024)
69	(2S)‐3‐*O*‐*β*‐D‐galactopyranose‐2‐*O*‐linolenoyl‐1‐*O*‐palmitoyl glycerol	LF	Zhang, Dai, et al. (2024)
70	(2S)‐3‐*O*‐*β*‐D‐galactopyranose‐2‐*O*‐linoleoyl‐1‐*O*‐palmitoyl glycerol	LF	Zhang, Dai, et al. (2024)
71	(2S)‐3‐*O*‐*β*‐D‐galactopyranose‐2‐*O*‐oleoyl‐1‐*O*‐palmitoyl glycerol	LF	Zhang, Dai, et al. (2024)
72	1‐*O*‐(9Z,12Z‐octadecadienoyl)‐2‐*O*‐(9Z,12Z,15Z‐octadecatrienoy‐3‐*O*‐*β*‐D‐galactopyranosyl glycerol)	LF	Zhou et al. 2022
73	*Lycium barbarum* polysaccharide‐I (LBP‐I)	LL	Zhao (2020)
74	*Lycium barbarum* polysaccharide‐II (LBP‐II)	LL	Zhao (2020)
75	*Lycium barbarum* polysaccharide‐III (LBP‐III)	LL	Zhao (2020)
76	*Lycium barbarum* polysaccharide‐IV (LBP‐IV)	LL	Zhao (2020)
77	*Lycium barbarum* leaves polysaccharide 5‐A (LBLP5‐A)	LL	Zhao (2020)
78	*Lycium barbarum* polysaccharide_3p_ (LBP_3p_)	LF	Wei et al. (2018)
79	*Lycium barbarum* glycopeptide‐1 (Lb Gp1)	LF	Wei et al. (2018)
80	*Lycium barbarum* glycopeptide‐2 (Lb Gp2)	LF	Wei et al. (2018)
81	*Lycium barbarum* glycopeptide‐3 (Lb Gp3)	LF	Wei et al. (2018)
82	*Lycium barbarum* glycopeptide‐4 (Lb Gp4)	LF	Wei et al. (2018)
83	*Lycium barbarum* glycopeptide‐5 (Lb Gp5)	LF	Wei et al. (2018)
84	*Lycium barbarum* polysaccharide fraction 1 (LBPF1)	LF	Wei et al. (2018)
85	*Lycium barbarum* polysaccharide fraction 2 (LBPF2)	LF	Wei et al. (2018)
86	*Lycium barbarum* polysaccharide fraction 3 (LBPF3)	LF	Wei et al. (2018)
87	*Lycium barbarum* polysaccharide fraction 4 (LBPF4)	LF	Wei et al. (2018)
88	*Lycium barbarum* polysaccharide fraction 5 (LBPF5)	LF	Wei et al. (2018)
89	*Lycium barbarum* polysaccharide A_3_ (LBPA_3_)	LF	Wei et al. (2018)
90	*Lycium barbarum* polysaccharide B_1_ (LBPB_1_)	LF	Wei et al. (2018)
91	*Lycium barbarum* polysaccharide C_2_ (LBPC_2_)	LF	Wei et al. (2018)
92	*Lycium barbarum* polysaccharide C_4_ (LBPC_4_)	LF	Wei et al. (2018)
93	*Lycium barbarum* neutral polysaccharide (LBNP)	LF	Wei et al. (2018)
94	LICP0093F‐2a	LF	Zeng et al. (2024)
95	*Lycium barbarum* polysaccharide 1B‐S‐2 (LBP1B‐S‐2)	LF	Zeng et al. (2024)
96	*Lycium barbarum* polysaccharide 1A1‐1 (LBP1A1–1)	LF	Zeng et al. (2024)
97	*Lycium barbarum* polysaccharide 1C‐2 (LBP1C‐2)	LF	Sun et al. (2023)
98	*Lycium barbarum* leaves polysaccharide 30 (LLP30)	LL	Quan (2018)
99	*Lycium barbarum* leaves polysaccharide 50 (LLP50)	LL	Quan (2018)
100	*Lycium barbarum* leaves polysaccharide 70 (LLP70)	LL	Quan (2018)
101	*Lycium barbarum* leaves polysaccharidet t (LLPt)	LL	Quan (2018)
102	*Lycium* ruthenicum leaves polysaccharide 1‐A (LRLP1‐A)	LL	Y. Liu (2016)
103	*Lycium ruthenicum* leaves polysaccharide 2‐A (LRLP2‐A)	LL	Y. Liu (2016)
104	*Lycium ruthenicum* leaves polysaccharide 3 (LRLP3)	LL	Y. Liu (2016)
105	*Lycium ruthenicum* leaves polysaccharide 4‐A (LRLP4‐A)	LL	Y. Liu (2016)
106	*Lycium ruthenicum* leaves polysaccharide 5‐A(LRLP5‐A)	LL	Y. Liu (2016)
107	*Lycium barbarum* polysaccharides‐2 (LBPs‐2)	LF	Zeng (2022)
108	*Lycium barbarum* polysaccharides‐3 (LBPs‐3)	LF	Zeng (2022)
109	1‐*O*‐(9Z,12Z,I5Z‐octadecatrienoyl)‐2‐*O*‐(9Z,12Z,15Z‐octadecatrienoyl)‐3‐*O*‐*β*‐D‐galactopyranosyl glycerol	LF	Zhou et al. (2022)
110	Naringenin‐7‐*O*‐rutinoside‐7‐*O*‐hexglycoside	LF	Zhang, Dai, et al. (2024)
**Alkaloids**			
111	Kukoamine A	LB	Lu et al. (2021)
112	Kukoamine B	LB	Lu et al. (2021)
113	Lyciumamide G	LB	Lu et al. (2021)
114	(E)‐2‐(4,5‐dihydroxy‐2‐{3‐[(4‐hydroxyphenethyl)amino]‐3‐oxopropyl}phenyl)‐3‐(4‐hydroxy‐3‐methoxyphenyl)‐nitrogen‐(4‐hydroxyphenethyl) acrylamide	LB	Lu et al. (2021)
115	(E)‐2‐(4,5‐dihydroxy‐2‐{3‐[(4‐hydroxyphenethyl)amino]‐3‐oxopropyl}phenyl)‐3‐(4‐hydroxy‐3,5‐dimethoxyphenyl)‐nitrogen‐(4‐hydroxyphenethyl) acrylamide	LB	Lu et al. (2021)
116	(E)‐2‐(4,5‐dihydroxy‐2‐{3‐[(4‐hydroxyphenethyl)amino]‐3‐oxopropyl}phenyl)‐3‐(4‐hydroxy‐3,5‐dimethoxyphenyl)‐nitrogen‐(4‐acetylamino butyl) acrylamide	LB	Lu et al. (2021)
117	(E)‐2‐(4,5‐dihydroxy‐2‐{3‐[(4‐hydroxyphenethyl)amino]‐3‐oxopropyl}phenyl)‐3‐(4‐hydroxy‐3‐ methoxyphenyl)‐nitrogen‐(4‐acetylamino butyl) acrylamide	LB	Lu et al. (2021)
118	Cannabisin F	LB	Lu et al. (2021)
119	Cannabisin D	LB	Lu et al. (2021)
120	Lyciumamide J	LB	Lu et al. (2021)
121	(1,2‐trans)‐nitrogen‐3‐(4‐acetylamino butyl)‐1‐(3,4‐dihydro‐xyphenethyl)‐7‐hydroxy‐dinitrogen‐(4‐hydroxyphenethyl)‐6,8‐dimethoxy‐1,2‐dihydronaphthalene‐2,3‐dimethylformamide	LB	Lu et al. (2021)
122	1,2‐dihydro‐6,8‐dimethoxy‐7‐hydroxy‐1‐(3,4‐dihydroxyphenethyl)‐itrogen‐1,2‐di‐[2‐(4‐hydroxyphenethyl)ethyl]‐2,3‐ naphthalenedicarboxamide	LB	Lu et al. (2021)
123	1,2‐ihydro‐6,8‐dimethoxy‐7‐hydroxy‐1‐(3,5‐dimethoxy‐4‐hydroxyphenethyl)‐nitrogen 1,2‐di‐[2‐(4‐hydroxyphenethyl)ethyl]‐2, 3‐naphthalenedicarboxamide	LB	Lu et al. (2021)
124	Melongenamide A	LF; LB	Lu et al. (2021)
125	1,2‐dihydro‐6,8‐dimethoxy‐7‐hydroxy‐1‐(3,4‐dihydroxyphenyl)‐N1, N2‐bis[2‐(4‐hydroxyphenyl) ethyl]‐2,3‐naphthalenedi carboxamide	LB	Lu et al. (2021)
126	(3S,4R)‐6‐hydroxy‐4‐(4‐hydroxy‐3,5‐dimethoxyphenyl)‐3‐(hydroxymethyl)‐N‐(4‐hydroxyphenethyl)‐5,7‐dimethoxy‐3,4‐dihydronaphthalene‐2‐carboxamide	LB	Lu et al. (2021)
127	Lyciumamide A	LF; LB	Zhou et al. (2022); Lu et al. (2021)
128	Cis‐N‐caffeoyltyramine	LB	Lu et al. (2021)
129	Cis‐N‐p‐hydroxyphenethyl ferulamide	LB	Lu et al. (2021)
130	N‐trans‐feruloyltyramine	LF; LB	Zhou et al. (2022); Lu et al. (2021)
131	3‐(4‐hydroxy‐3‐methoxyphenyl)‐N‐[2‐(4‐hydroxyphenyl)‐2‐methoxyethyl]acrylamide	LB	Lu et al. (2021)
132	N‐trans‐cinnamoyl tyramine	LB	Lu et al. (2021)
133	N‐trans‐p‐hydroxycoumaroyl tyramine	LB	Lu et al. (2021)
134	N‐trans‐p‐hydroxyphenethyl caffeamide	LB	Lu et al. (2021)
135	N‐trans‐coumaroyloctopamine	LB	Lu et al. (2021)
136	N‐[2‐(3,4‐dih‐ydroxyphenyl)‐2‐hydroxyethyl]‐3‐(4‐methoxyphenyl) prop‐2‐enamide	LB	Lu et al. (2021)
137	N‐trans‐feruloyl‐3′‐*O*‐methyldopamine	LB	Lu et al. (2021)
138	N‐trans‐sinapoyltyramine	LB	Lu et al. (2021)
139	(2S,E)‐N‐[2‐hydroxy‐2‐ (4‐hydroxyphenyl) ethyl ester] ferulamide	LB	Lu et al. (2021)
140	N‐trans‐caffeoyltyramine	LB	Lu et al. (2021)
141	N‐trans‐feruloyl demethylated sinomenine	LB	Lu et al. (2021)
142	Lyciumide A	LB	Lu et al. (2021)
143	Dihydro‐feruloyltyramine	LB	Lu et al. (2021)
144	Dihydro‐caffeoyltyramine	LB	Lu et al. (2021)
145	Dihydroferuloyl‐5‐methoxytyramine	LB	Lu et al. (2021)
146	N‐acetyl‐N′‐trans‐feruloylputrescine	LB	Lu et al. (2021)
147	Lyciumamide	LB	Lu et al. (2021)
148	Cannabisin G	LB	Lu et al. (2021)
149	(E)‐3‐{(2,3‐trans)‐2‐(4‐Hydroxy‐3‐methoxyphenyl)‐3‐hydroxy‐methyl‐2,3‐dihydrobenzo[b][1,4]dioxin‐6‐yl}‐N‐(4‐hydroxyphenethyl) acrylamide	LB	Lu et al. (2021)
150	(Z)‐3‐{(2,3‐trans)‐2‐(4‐Hydroxy‐3‐methoxyphenyl)‐3‐hydroxy‐methyl‐2,3‐dihydrobenzo[b][1,4]dioxin‐6‐yl}‐N‐(4‐hydroxy‐phenethyl) acrylamide	LB	Lu et al. (2021)
151	(2,3‐trans)‐3‐(3‐Hydroxy‐5‐methoxy‐phenyl)‐N‐(4‐hydroxyphenethyl)‐7‐{(E)‐3‐[(4‐hydroxyphenethyl)amino]‐3‐oxoprop‐1‐en‐1‐yl}‐2,3‐dihydrobenzo[b][1,4]dioxine‐2‐carboxamide	LB	Lu et al. (2021)
152	(2,3‐trans)‐3‐(3‐Hydroxy‐5‐methoxyphenyl)‐N‐(4‐hydroxy‐phenethyl)‐7‐{(Z)‐3‐[(4‐hydroxyphenethyl)amino]‐3‐oxoprop‐1‐en‐1‐yl}‐2,3‐dihydrobenzo[b][1,4]dioxine‐2‐carboxamide	LB	Lu et al. (2021)
153	Lyciumamide D	LB	Lu et al. (2021)
154	Lyciumamide E	LB	Lu et al. (2021)
155	(2S,3S,E)‐3‐{−2‐(4‐hydroxy‐3,5‐methoxyphenyl)‐3‐hydroxymethyl‐2,3‐dihydrobenzo[b][1,4]dioxin‐6‐yl}‐N‐(4‐hydroxyphenethyl)‐acrylamide	LB	Lu et al. (2021)
156	(2R,3R,E)‐3‐{−2‐(4‐hydroxy‐3,5‐methoxyphenyl)‐3‐hydroxymethyl‐2,3‐dihydrobenzo[b][1,4]dioxin‐6‐yl}‐N‐(4‐hydroxyphenethyl)‐acrylamide	LB	Lu et al. (2021)
157	Erythro‐canabisine H	LB	Lu et al. (2021)
158	Grossamide	LB	Lu et al. (2021)
159	Grossamide K	LB	Lu et al. (2021)
160	Dihydgrossamide	LB	Lu et al. (2021)
161	Grossamide	LB	Lu et al. (2021)
162	7‐hydroxy‐1‐(4‐hydroxy‐3‐methoxyphenyl)‐N2, N3‐bis‐(4‐hydroxyphenethyl)‐6‐methoxy‐1,2‐dihydronaphthalene‐2,3‐dicarboxamide	LB	Lu et al. (2021)
163	Calystegine A3	LB	Lu et al. (2021)
164	Calystegine A5	LB	Lu et al. (2021)
165	Calystegine A6	LB	Lu et al. (2021)
166	Calystegine A7	LB	Lu et al. (2021)
167	Calystegine B1	LB	Lu et al. (2021)
168	Calystegine B2	LB	Lu et al. (2021)
169	Calystegine B3	LB	Lu et al. (2021)
170	Calystegine B4	LB	Lu et al. (2021)
171	Calystegine B5	LB	Lu et al. (2021)
172	Calystegine C1	LB	Lu et al. (2021)
173	Calystegine C2	LB	Lu et al. (2021)
174	Calystegine N1	LB	Lu et al. (2021)
175	N‐methyl‐calystegine B2	LB	Lu et al. (2021)
176	N‐methyl‐calystegine C1	LB	Lu et al. (2021)
177	Atropine	LL; LB	Zhang, Dai, et al. (2024); Harsh (1989)
178	Scopolamine	LL; LB	Zhang, Dai, et al. (2024); Harsh (1989)
179	Hyoscyamine	LF; LB	Zhang, Dai, et al. (2024); Harsh (1989)
180	Fagomine	LB	Lu et al. (2021)
181	6‐Deoxy fagomine	LB	Lu et al. (2021)
182	N‐feruloyltyramine dimer	LF	Zhou et al. (2022)
183	Methyl‐2‐[2‐formyl‐5‐(methoxymethyl)‐1H‐pyrrol‐1‐yl]‐3‐ (4‐hydroxyphenyl)propanoate	LF	Zhou et al. (2022)
184	Dimethyl‐2‐[2‐formyl‐5‐(methoxymethyl)‐1H‐pyrrol‐1‐yl]butanedioate	LF	Zhou et al. (2022)
185	Methyl‐2‐[2‐formyl‐5‐(methoxymethyl)‐1H‐pyrrol‐1‐yl] propanoate	LF	Zhou et al. (2022)
186	Lycii‐alkaloid‐i	LF	Zhou et al. (2022)
187	Lycii‐alkaloid‐iii	LF	Zhou et al. (2022)
188	Lycii‐alkaloid‐iv	LF	Zhou et al. (2022)
189	Betaine	LF; LL; LB	Zhao (2020); Yan et al. (2024); Lu et al. (2021)
190	Choline	LF; LB	Zhang, Dai, et al. (2024)
191	Allantoin	LB	Lu et al. (2021)
192	Vernine	LB	Lu et al. (2021)
193	2‐furylcarbinol‐(5′‐11)‐1,3‐cyclo⁃pentadiene‐[5,4‐c]‐1H‐cinnoline	LF	Zhou et al. (2022)
194	Lycioside A	LF	Zhou et al. (2022)
195	Lycioside B	LF	Zhou et al. (2022)
**Phenolics**			
196	Cis‐p‐Hydroxybenzoic acid	LF; LB	Zhang, Dai, et al. (2024); Lu et al. (2021)
197	Tran‐p‐Hydroxybenzoic acid	LF	Zhang, Dai, et al. (2024)
198	Chlorogenic acid	LF; LL	Zhang, Dai, et al. (2024); Zhao (2020)
199	Cryptochlorogenic acid	LF; LL	Zhang, Dai, et al. (2024); Zhao (2020)
200	Isochlorogenic acid A	LF	Zhang, Dai, et al. (2024)
201	Isochlorogenic acid B	LF	Zhang, Dai, et al. (2024)
202	Isochlorogenic acid C	LF	Zhang, Dai, et al. (2024)
203	Protocatechuate	LF	Zhang, Dai, et al. (2024)
204	p‐coumaric acid	LF; LL	Zhang, Dai, et al. (2024); Ma (2024b)
205	p‐hydroxybenzoic acid	LF; LL	Zhang, Dai, et al. (2024); Zhao (2020)
206	Neochlorogenic acid	LF; LL	Zhang, Dai, et al. (2024); Zhao (2020)
207	3,4‐Dihydroxybenzaldehyde	LL	Zhao (2022)
208	Caffeic acid	LF; LL; LB	Zhang, Dai, et al. (2024); Lu et al. (2021); Song et al. (2024)
209	Trans‐Sinapinic acid	LF	Ma et al. (2024)
210	Ferulic acid	LF; LL; LB	Zhang, Dai, et al. (2024); Zhao (2020); Lu et al. (2021)
211	Gentisic acid	LF; LL	Zhou et al. (2022); Song et al. (2024)
212	Vanillic acid	LF; LL	Zhou et al. (2022); Song et al. (2024)
213	Phthalic acid	LL	Song et al. (2024)
214	4‐Hydroxyphenylacetic acid	LL	Song et al. (2024)
215	3,4‐dihydroxyphenylacetic acid	LL	Song et al. (2024)
216	3‐ (4‐hydroxyphenyl) lactic acid	LL	Song et al. (2024)
217	4‐Hydroxycinnamic acid	LL; LB	Lu et al. (2021); Song et al. (2024)
218	3,4‐dihydroxyphenylpropionic acid	LB	Lu et al. (2021)
219	4‐*O*‐*β*‐D‐Glucopyranosyl‐cis‐p‐coumaric acid	LF	Ma et al. (2024)
220	4‐*O*‐*β*‐D‐Glucopyranosyl‐cis‐ferulic acid	LF	Ma et al. (2024)
221	4‐*O*‐*β*‐D‐Glucopyranosyl‐trans‐caffeic acid	LF	Ma et al. (2024)
222	4‐*O*‐(*β*‐D‐Glucopyranosyl‐4‐*β*‐D‐glucopyranosyl)‐trans‐ferulic acid	LF	Ma et al. (2024)
223	3‐*O*‐*β*‐D‐Glucopyranosyl‐trans‐caffeic acid	LF	Ma et al. (2024)
224	4‐*O*‐*β*‐D‐Glucopyranosyl‐trans‐p‐coumaric acid	LF	Ma et al. (2024)
225	Proanthocyanins B1	LF	Zhang, Dai, et al. (2024)
226	Proanthocyanins B2	LF	Zhang, Dai, et al. (2024)
** *Lycium barbarum* pigment**			
227	*β*‐Carotene	LF	Zhang, Dai, et al. (2024); Su et al. (2024); Li et al. (2006)
228	Zeaxanthin	LF	Zhang, Dai, et al. (2024); Su et al. (2024); Li et al. (2006)
229	Lutein	LF	Zhang, Dai, et al. (2024); Su et al. (2024)
230	*β*‐cryptoflavin	LF	Zhang, Dai, et al. (2024); Su et al. (2024)
231	Neoxanthin	LF	Zhang, Dai, et al. (2024)
232	Zeaxanthin monomicronate	LF	Su et al. (2024)
233	Zeaxanthin monopalmitate	LF	Su et al. (2024); Inbaraj et al. (2008)
234	Zeaxanthin dipalmitate	LF	Su et al. (2024); Li et al. (2006)
235	*β*‐cryptoflavin monopalmitate	LF	Su et al. (2024); Inbaraj et al. (2008)
236	Violaxanthin dipalmitate	LF	Su et al. (2024)
237	Antheraxanthin dipalmitate	LF	Su et al. (2024)
238	Malvidin‐3,5‐diglucoside	LF	Zhang, Dai, et al. (2024)
239	Delphinidin‐3‐*O*‐glucoside	LF	Zhang, Dai, et al. (2024)
240	Petunidin‐5‐*O*‐glucoside	LF	Zhang, Dai, et al. (2024)
241	Peonidin‐3‐*O*‐glucoside	LF	Zhang, Dai, et al. (2024)
242	Malvidin‐3‐*O*‐(6‐*O*‐p‐ocoumaroyl)glucoside	LF	Zhang, Dai, et al. (2024)
243	Delphinidin‐3‐*O*‐(6‐*O*‐acetyl)glucoside	LF	Zhang, Dai, et al. (2024)
244	Petunidin‐3‐*O*‐glucoside	LF	Zhang, Dai, et al. (2024)
245	Petunidin‐3‐*O*‐rutinoside‐5‐*O*‐glucoside	LF	Zhang, Dai, et al. (2024)
246	Petunidin‐3‐*O*‐galactoside‐5‐*O*‐glucoside	LF	Zhang, Dai, et al. (2024); Gan et al. (2021)
247	Petunidin‐3‐*O*‐(cis‐p‐ocoumaroyl) rutinoside‐5‐*O*‐glucoside	LF	Zhang, Dai, et al. (2024); Gan et al. (2021)
248	Petunidin‐3‐*O*‐(trans‐p‐ocoumaroyl) rutinoside‐5‐*O*‐glucoside	LF	Zhang, Dai, et al. (2024); Gan et al. (2021)
249	Delphinidin‐3‐*O*‐rutinoside(p‐ocoumaroyl)‐5‐*O*‐glucoside	LF	Zhang, Dai, et al. (2024); Gan et al. (2021)
250	Petunidin‐3‐*O*‐rutinoside(cis‐p‐ocoumaroyl)‐5‐*O*‐glucoside	LF	Zhang, Dai, et al. (2024); Gan et al. (2021)
251	Petunidin‐3‐*O*‐rutinoside(trans‐p‐ocoumaroyl)‐5‐*O*‐glucoside	LF	Zhang, Dai, et al. (2024), Gan et al. (2021)
252	Petunidin‐3‐*O*‐glucoside‐5‐*O*‐glucoside	LF	Zhang, Dai, et al. (2024); Gan et al. (2021)
253	Petunidin‐3‐*O*‐glucoside(feruloyl)‐5‐*O*‐glucoside	LF	Zhang, Dai, et al. (2024); Gan et al. (2021)
254	Malvidin‐3‐*O*‐(6‐*O*‐p‐ocoumaroyl‐3‐*O*‐acetyl)‐5‐*O*‐diglucoside	LF	Zhang, Dai, et al. (2024); Gan et al. (2021)
255	Petunidin‐3‐*O*‐rutinoside‐p‐ocoumaroyl	LF	Zhang, Dai, et al. (2024), Gan et al. (2021)
256	Delphinidin‐3‐*O*‐coumarylglucoside	LF	Zhang, Dai, et al. (2024)
257	Petunidin‐3‐*O*‐coumarylsophoroside‐5‐*O*‐ glucoside	LF	Zhang, Dai, et al. (2024)
258	Petunidin‐3‐*O*‐(6‐*O*‐p‐ocoumaroyl)‐rutinoside‐5‐*O*‐glucoside	LF	Zhang, Dai, et al. (2024)
259	Malvidin	LF	Zhang, Dai, et al. (2024)
260	Cyanidin	LF	Zhang, Dai, et al. (2024)
261	Pelargonidin	LF	Zhang, Dai, et al. (2024)
262	Peonidin	LF	Zhang, Dai, et al. (2024)
263	Delphinidin	LF	Zhang, Dai, et al. (2024)
264	Petunidin	LF	Zhang, Dai, et al. (2024)
**Amino acids**			
265	Threonine	LF; LL; LB	Lu et al. (2021); Yan et al. (2014)
266	Valine	LF; LL; LB	Lu et al. (2021); Yan et al. (2014); Liang et al. (2021)
267	Methionine	LF; LL; LB	Lu et al. (2021); Yan et al. (2014); Liang et al. (2021)
268	Isoleucine	LF; LL; LB	Lu et al. (2021); Yan et al. (2014)
269	Leucine	LF; LL; LB	Lu et al. (2021); Yan et al. (2014)
270	Phenylalanine	LF; LL; LB	Lu et al. (2021); Yan et al. (2014); Liang et al. (2021)
271	Lysine	LF; LL; LB	Lu et al. (2021); Yan et al. (2014); Liang et al. (2021)
272	Aspartic acid	LF; LL; LB	Lu et al. (2021); Wang, Shan, et al. (2020); Yan et al. (2014)
273	Serine	LF; LL; LB	Lu et al. (2021); Yan et al. (2014)
274	Glycine	LF; LL; LB	Lu et al. (2021); Yan et al. (2014); Liang et al. (2021)
275	Alanine	LF; LL; LB	Lu et al. (2021), Zhou et al. (2022); Yan et al. (2014)
276	Tyrosine	LF; LL; LB	Lu et al. (2021); Yan et al. (2014); Liang et al. (2021)
277	Histidine	LF; LL; LB	Lu et al. (2021); Yan et al. (2014)
278	Arginine	LF; LL; LB	Lu et al. (2021); Yan et al. (2014); Liang et al. (2021)
279	Proline	LF; LL; LB	Lu et al. (2021); Yan et al. (2014); Liang et al. (2021)
280	Cystine	LF; LL	Wang, Shan, et al. (2020); Yan et al. (2014)
281	Glutamic acid	LF; LL; LB	Lu et al. (2021); Yan et al. (2014); Liang et al. (2021)
282	Tryptophan	LF; LL; LB	Lu et al. (2021); Yan et al. (2014); Liang et al. (2021)
283	4‐aminobutyric acid	LL	Liang et al. (2021)
284	Citrulline	LL	Liang et al. (2021)
285	Ornithine	LL	Liang et al. (2021)

### 2.1 Flavonoids

**FIGURE 3:**
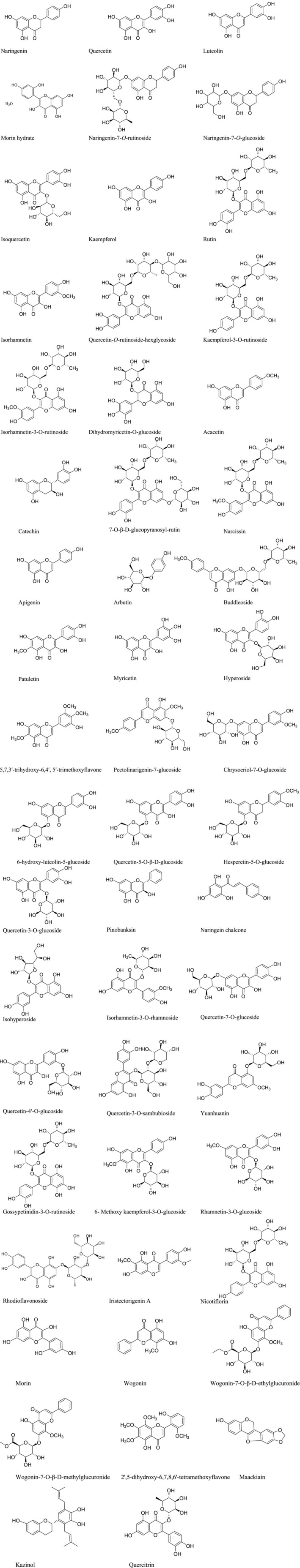
The chemical structure of flavonoids in *Lycium barbarum*.

Flavonoids are a complex and diverse group of secondary metabolites found in plants. Flavonoids are typically divided into seven subtypes according to their degree of oxidation and position of carbon bonding: flavonols, flavones, isoflavones, anthocyanins, flavanones, flavanols, and chalcones (Liu, et al. 2022). Flavonoids are among the primary chemical components of *Lycium barbarum* , serving as important quality *markers*. They are distributed in the fruit, leaves, and root bark of *Lycium barbarum* . The flavonoid content varies significantly among the different organs, tissues, and production areas of *Lycium barbarum* , with the highest concentration present in the leaves (Zhao 2020). Yan et al. used spectrophotometric colorimetry (Yan et al. 2024) and reported that the contents of flavonoids in LF and LL were 15.02–22.16 mg/g and 18.10–25.07 mg/g, respectively, which were higher than those (4.50–5.89 mg/g) in LB. In terms of different production areas, Ma used the aluminum nitrate‐sodium nitrite colorimetric method and reported that the total flavonoid content in LF from Ningxia was the highest (57.87 μg/g), followed by that in LF from Qinghai (50.77 μg/g) and Gansu (47.86 μg/g) (Ma 2024b). Li, Cui, and Zhang (2024) reported that the flavonoid content of goji berries from the Gansu production area was highest, followed by those from the Yellow River Delta and Shanxi. Additionally, Liu, Yang, et al. (2019); Liu, Long, et al. (2019) reported that as the storage time increased, the internal loss of LF increased, and the rutin content decreased, suggesting that the freshness of LF can be judged on the basis of the flavonoid extraction rate. Modern research has demonstrated that *Lycium barbarum* flavonoids are the primary basis for its pharmacological activities, including antioxidant, antiaging, and cardiovascular and cerebrovascular health benefits. Currently, at least 53 flavonoids, including naringenin, rutin, quercetin, and luteolin, have been identified in *Lycium barbarum* (Figure 3).

### 2.2 Polysaccharides

*Lycium barbarum* polysaccharides are water‐soluble complex polysaccharides that are primarily composed of rhamnose, galactose, glucose, mannose, xylose, galacturonic acid, and various amino acids or lipids (Zhang, Dai, et al. 2024). Similar to flavonoids, the polysaccharide content in *Lycium barbarum* is also influenced by different tissues and production areas. In terms of tissues, the polysaccharide contents in LF and LL are 2.74%–3.37% and 2.82%–3.53%, respectively, which are higher than the polysaccharide content (1.92%–2.68%) in LB (Yan et al. 2024). Moreover, in terms of polysaccharides in LL (132.25 mg/g), the average contents of neutral polysaccharides and acidic polysaccharides are comparable at 18.99 mg/g and 18.69 mg/g, respectively (Zhao 2020). In terms of the production area, the polysaccharide content in LF from Ningxia was the highest, followed by those from Ningxia and Gansu (Liu, Yang, et al. (2019); Liu, Long, et al. (2019)). Polysaccharides are highly active and complex, and their content is an important criterion for determining the quality of *Lycium barbarum* . It is also a key factor in the deep processing, comprehensive development, and utilization of *Lycium barbarum* .

### 2.3 Alkaloids

**FIGURE 4:**
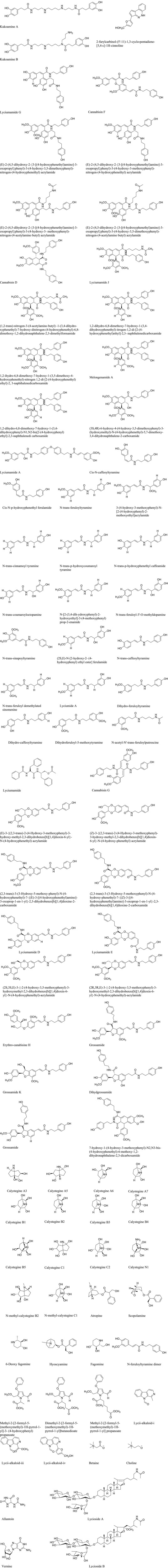
The chemical structure of alkaloids in *Lycium barbarum*.

Alkaloids are a class of nitrogen‐containing organic compounds present in various parts of *Lycium barbarum* , with the greatest number of compounds observed in LBs (Lu et al. 2021). Alkaloids, including amide alkaloids, calystegine alkaloids, tropine alkaloids, piperidine alkaloids, pyrrole alkaloids, carboline alkaloids, and other alkaloids, are among the major characteristic components of *Lycium barbarum* (Zhou et al. 2022) (Figure 4). Research has demonstrated that betaine is present in LL, LF, and LB; however, its content varies. The content of betaine in LL was the highest, ranging from 3.92% to 6.55% in mass fraction. The content in LF was the second highest, with a mass fraction of 1%–2.8%, while the lowest content was found in LB, with a mass fraction of 0.5%–1% (Ma 2024b). *Lycium barbarum* alkaloids, including choline, betaine, and hyoscyamine, exhibit various biological activities.

### 2.4 Phenolics

**FIGURE 5:**
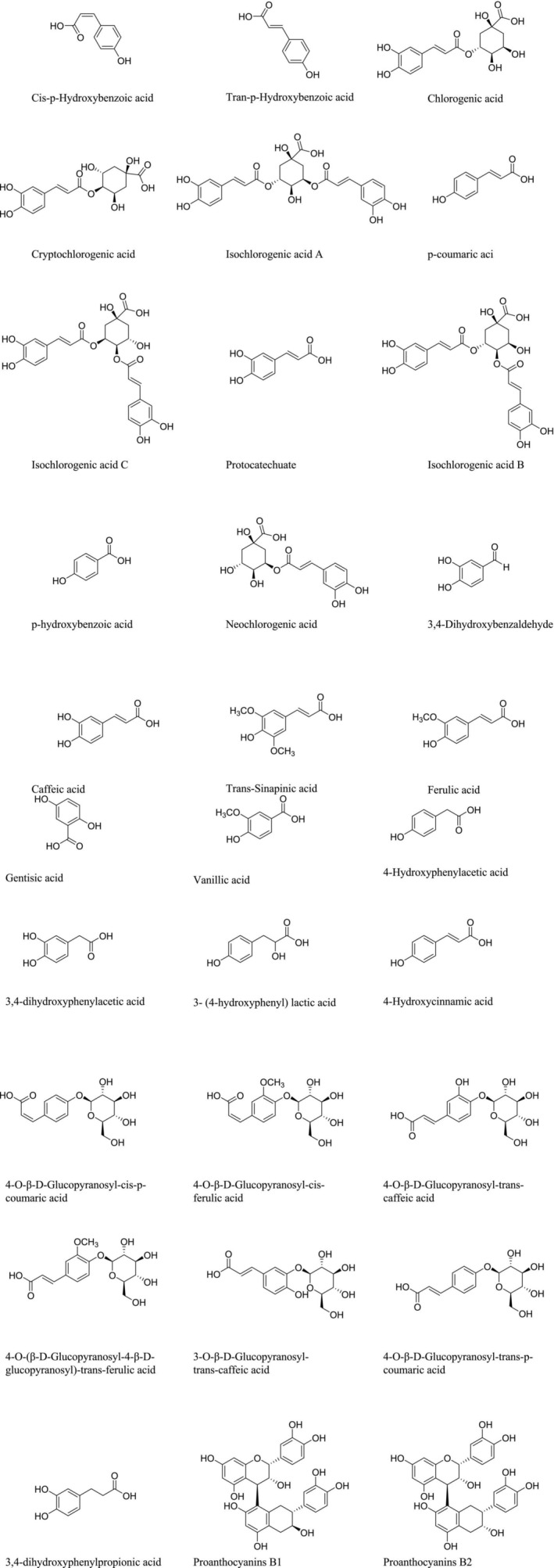
The chemical structure of phenolics in *Lycium barbarum*.

Phenolics are aromatic compounds in which a hydroxyl group substitutes for the hydrogen of the benzene ring, and they are secondary metabolites in plants. Phenolic acids are among the major nutritional and functional components involved in the prevention of cardiovascular diseases, tumors, and metabolic diseases, such as diabetes and gout (Zhou et al. 2022). Currently, 31 phenolic compounds belonging to the class of phenylpropanoids have been identified in *Lycium barbarum* (Figure 5). Inbaraj et al. (2010) reported that chlorogenic acid, p‐coumaric acid, and caffeic acid are phenolic acids present at high levels in *Lycium barbarum* on the basis of high‐performance liquid chromatography‐diode array detection‐electrospray ionization tandem mass spectrometry. Bondia‐Pons et al. (2024) reported that caffeic acid derivatives and coumaric acid derivatives are the major phenolic acid components of *Lycium barbarum* , as determined by high‐performance liquid chromatography tandem quadrupole time‐of‐flight mass spectrometry. Notably, although there are no comparative studies on the phenolic content in LL, LF, and LB, relevant studies have shown that the phenolic content in wolfberry fruits ranges from 121.588 to 191.457 mg/g (Ma 2024b). Lü et al. reported that the phenolic content in wolfberry leaves is affected by their location, and overall, the phenolic content in the leaves of the shoot (approximately 30 mg/g) is greater than that in the leaves of the base (approximately 26 mg/g) (Lv 2021).

### 2.5 *Lycium barbarum* Pigment

**FIGURE 6:**
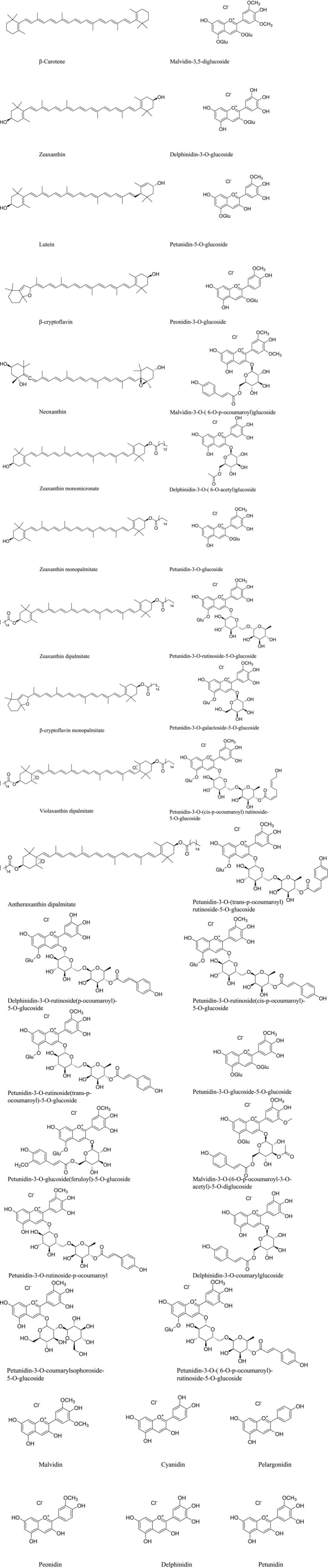
The chemical structure of *Lycium barbarum* pigment in *Lycium barbarum*.

*Lycium barbarum* pigment is a general term for various coloring substances in *Lycium barbarum* that are primarily composed of carotenoids and their esters (227–237), anthocyanins (238–258), and anthocyanidin (259–264), which exhibit pharmacological activities, such as antioxidant, eye protection, liver protection, and tumor prevention activities (Su et al. 2024) (Figure 6). Inbaraj et al. (2008) reported that the highest content of carotenoids and their ester compounds in *Lycium barbarum* was in the form of eaxanthin dipalmitate (1143.7 μg/g), followed by *β*‐cryptoflavin monopalmitate and its two isomers (32.9–68.5 μg/g), zeaxanthin and monopalmitat, and its two isomers (11.3–62.8 μg/g), all trans‐β‐carotene (23.7 μg/g) and all trans‐zeaxanthin (1.4 μg/g). Li et al. (2006) reported that the total carotenoid content of *Lycium barbarum* increases continuously throughout the growth process and that the composition changes primarily toward zeaxanthin dipalmitate. Anthocyanins are a class of water‐soluble pigments formed by the combination of anthocyanins with glucose, rhamnose, and arabinose via glycosidic bonds, including malvidin‐3,5‐diglucoside, delphinidin‐3‐*O*‐glucoside, and petunidin‐5‐*O*‐glucoside (Zhang, Dai, et al. 2024; Gan et al. 2021). *Lycium barbarum* pigments exhibit the physiological and pharmacological activities of natural edible pigments and can be used as a natural coloring agent as well as for coloring food and cosmetics.

### 2.6 Amino Acids

Amino acids are the major nutritional and medicinal components of *Lycium barbarum* and are present in LF, LL, and LB. Ji et al. (2022) reported that under organic cultivation, LF contains 16 amino acids with a total content of 75.13 g/kg. The proline content was the highest (13.78 g/kg), and the methionine content was the lowest (0.17 g/kg). Lu et al. (2021) reported that LB contains 17 amino acids, including methionine and valine. Wang, Zhang, et al. (2020) reported that LL contains 18 amino acids. Within the 1–4 year planting period, the essential amino acid and total amino acid contents under LL decreased with increasing years of cultivation. The content of essential amino acids in annual LL was 2.18 g/100 g, whereas the content of essential amino acids in 4‐year LL was only 63% of that in annual LL. The total amino acid content in annual LL was as high as 7.98 g/100 g, whereas the total amino acid content in 4‐year LL decreased to 5.23 g/100 g. Yan et al. (2014) reported that both LL and LF contain 18 amino acids, including 10 essential and 8 nonessential amino acids, with the highest content of both essential and nonessential amino acids in the leaves. *Lycium barbarum* is an important medicinal and edible plant in China. Its amino acids not only possess physiological activities to promote metabolism and improve liver and heart function, but also serve as an important basis for measuring food quality. Its content is closely related to the nutrition and taste of food and is important for the development of *Lycium barbarum* products.

## 3 Pharmacological Effects

### 3.1 Antidiabetic Effects of *Lycium barbarum*

The mortality rate of diabetes mellitus (DM) is the third highest after that of malignant tumors and cardiovascular and cerebrovascular diseases, which seriously threaten human health (Peng et al. 2024). *Lycium barbarum* , a medicinal and food homologous product, is rich in various components, such as flavonoids, polysaccharides, alkaloids, and phenolic acids, that exert good hypoglycemic effects (Zhu et al. 2013). It can play a therapeutic role in diabetes by improving glucose metabolism, acting as an antioxidant, regulating intestinal flora, inhibiting the activities of α‐amylase and dipeptidyl peptidase IV (DPP‐4), and exerting other beneficial effects.

#### 3.1.1 *Lycium barbarum* Fruit and Its Extracts

LF polysaccharides can reduce fasting blood glucose (FBG), insulin (INS), the insulin resistance index (IRI), serum total triglyceride (TG), total cholesterol (TC), interleukin‐18 (IL‐18), IL‐6, malonic dialdehyde (MDA), endometrial ACSL4, and PTGS2 protein expression levels; increase serum superoxide dismutase (SOD); and play a protective role in gestational DM rats. Its mechanism of action is related to the promotion of nuclear factor erythroid 2‐related factor 2 (Nrf2)/heme oxygenase‐1 (HO‐1)/glutathione peroxidase 4 (GPX4) signal transduction, reduction in oxidative stress, inhibition of the iron death pathway, and enhancement of insulin sensitivity (Zhang, Xie, and Sun 2024). LF polysaccharides can also reduce water consumption, body weight, FBG, and INS levels in diabetic rats induced by a high‐fat diet combined with streptozotocin (STZ), increase the activity of endothelial nitric oxide synthase (eNOS) and the content of nitric oxide (NO), and improve glucose tolerance and vascular endothelium‐dependent relaxation function in rats by inhibiting oxidative stress, thereby reducing damage to pancreatic beta cells (Guo et al. 2024; Chen et al. 2023). Inhibiting the activity of α‐glucosidase can delay and control the digestion and absorption of glucose in the small intestine. Inhibiting the activity of α‐amylase can weaken the rate of starch breakdown in the human digestive tract. Therefore, inhibiting the activity of α‐amylase and α‐glucosidase plays a crucial role in reducing postprandial blood glucose levels. In vitro studies have demonstrated that LF polysaccharides can significantly inhibit the activities of α‐amylase and α‐glucosidase. When the polysaccharide concentration is 6 mg/mL, the inhibition rates of α‐amylase and α‐glucosidase activities reach 92.59% ± 0.47% and 92.09% ± 0.21%, respectively (Guo and Dong 2024). It is also possible to regulate blood glucose levels by reducing glucose absorption in the human intestinal epithelial cell line Caco‐2 and inhibiting the expression of sodium glucose cotransporter 1 (SGLT‐1) (Cai et al. 2017). The total flavonoids in LF can reduce body weight, FBG, and blood glucose levels at 30, 60, and 120 min after glucose loading in alloxan‐induced DM mice, with a significant hypoglycemic effect that occurs in a concentration‐dependent manner (Zhang, Lin, et al. 2022). The water extract of LF can reverse weight loss, increase FBG, and abnormal feeding and drinking conditions in alloxan‐induced DM mice and can also inhibit the increase in blood sugar levels according to the glucose tolerance test. Its hypoglycemic effect is related to a reduction in the level of MDA in the serum and improvements in the activities of SOD and glutathione peroxidase (GSH‐Px) (H. Liu 2020).

#### 3.1.2 *Lycium barbarum* Leaf and Its Extracts

LL polysaccharide can not only inhibit the activities of α‐amylase and α‐glucosidase (Quan et al. 2023) but also improve the symptoms of polydipsia, overeating, and weight loss in type I DM mice caused by STZ, reduce the levels of FBG and serum glycosylated hemoglobin, and increase the activities of the catalyst (CAT) and total superoxide dismutase (T‐SOD) in the serum and liver homogenate (Quan 2018). The LL extract, which contains neochlorogenic acid, chlorogenic acid, caffeic acid, and rutin, significantly reduced the levels of INS, TC, TG, LDL‐C, and free fatty acid (FFA) in the serum of type 2 DM rats; repaired damage to the glomeruli, renal tubules, and islet cells; and played a protective role in type 2 DM rats induced by a high‐fat diet combined with STZ. Its mechanism is related to improving metabonomics and increasing intestinal flora diversity (Zhao, Guo, et al. 2020; Zhao, Xiao, et al. 2020). The methanol extract of LL can inhibit α‐amylase activity in a concentration‐dependent manner, with particular inhibitory effects observed for chlorogenic acid, salicylic acid, and caffeic acid on α‐amylase (Pollini et al. 2020). The ethyl acetate extract of LL reduced FBG levels; altered lipid profiles; reduced the activities of alkaline phosphatase (ALP), aspartate transaminase (AST), and alanine transaminase (ALT); elevated the antioxidant status of SOD, CAT, and GSH‐Px; and reduced MDA levels, exerting a protective effect on STZ‐induced diabetic rats (Olatunji et al. 2017). Wei et al. (2012) reported that LL decoction, administered in all seasons, could reduce FBG levels in alloxan‐induced diabetic mice and improve symptoms of polydipsia, overeating, polyuria, emaciation, decreased activity, and mental depression. When the dose of the LL decoction was 750 mg/kg, the hypoglycemic rates of LL during the spring, summer, and autumn harvesting periods were 31.67%, 24.91%, and 20.62%, respectively, and these rates were similar to the hypoglycemic rate of metformin hydrochloride (27.71%). Ma (2024b) reported that polyphenols, alkaloids, polysaccharides, and proteins in LL can significantly inhibit the activity of α‐glucosidase and α‐amylase, thereby increasing postprandial blood glucose levels. Among these, the half‐maximal inhibitory concentrations (IC_50_) of polyphenols, alkaloids, polysaccharides, and proteins in LL for α‐glucosidase were 1.248 mg/mL, 8.290 mg/mL, 9.530 mg/mL, and 9.884 mg/mL, respectively. The IC_50_ values for α‐amylase were 1.103, 1.431, 23.81, and 21.092 mg/mL, respectively. LL and its extracts have been observed to play a role in the prevention and treatment of DM by inhibiting enzyme activity, acting as antioxidants, and regulating intestinal flora.

#### 3.1.3 *Lycium barbarum* Root Bark and Its Extracts

The extract of LB, which contains chlorogenic acid, scopoletin, kukoamine B, and kukoamine A, can significantly inhibit the activity of α‐glucosidase. The IC_50_ values of LB extract harvested at 1, 3, 5, and 7 years were 73.2, 35.4, 32.8, and 32.3 mg/mL, respectively, indicating that the ability to inhibit α‐glucosidase activity gradually increases with increasing growth duration (Li 2023). The water LB extract significantly reduced the levels of FBG, TG, TC, low‐density fatty acids (LDL‐C), and FFAs; increased the level of high‐density fatty acids (HDL‐C); and reduced the heterotopic deposition of liver lipids in insulin‐resistant rats with type 2 DM, thereby improving the metabolism of glucose and lipids and reducing the extent of hepatic adipocyte damage. Its mechanism is related to the upregulation of glucose transporter 4 (GLUT4) and peroxisome proliferator‐activated receptor α protein levels, the downregulation of glycogen synthase kinase 3*β* (GSK3*β*) protein levels, and the improvement of IR through the AMPK signaling pathway (Yan and Liu 2011; Yao et al. 2020). Decoction of LB can improve the symptoms of polydipsia, overeating, polyuria, emaciation, decreased activity, and mental depression; inhibit the production of oxygen free radicals in the body; increase antioxidant capacity; accelerate the clearance of free radicals; and reduce pancreatic islet damage (Huang et al. 2018; Wei et al. 2009). Additionally, a decoction of LB dose‐dependently reduced blood glucose levels in two types of hyperglycemic rats induced by streptozotocin and adrenaline hydrochloride (Cui et al. 2016). Fang et al. (2004) reported that LB decoction significantly increased the content of serum INS and liver glycogen in alloxan‐induced DM rats, reduced FBG levels, improved the function of pancreatic islets, promoted the synthesis of liver glycogen, and reduced blood sugar levels in normal rats.

### 3.2 Antioxidant Effects of *Lycium barbarum*

The bioactive components with antioxidant effects reported in recent years are primarily polysaccharides, alkaloids, phenolics, and flavonoids, which are abundant in various parts of *Lycium barbarum* (Liu, Liu, et al. 2022; Sun et al. 2023; Bondia‐Pons et al. 2024).

#### 3.2.1 *Lycium barbarum* Fruit and Its Extracts

In vitro experiments demonstrated that at a mass concentration of 350 μg/mL, the DPPH free radical scavenging rate of LF polyphenols reached 80.4% of that of vitamin C, and the ABTS free radical scavenging rate reached 94.3% of that of vitamin C (Yin et al. 2024). LF polysaccharides can effectively scavenge free radicals such as O^−2^, 1,1‐diphenyl‐2‐ (DPPH), and OH, and their clearance efficiency is positively correlated with their concentration (Gao et al. 2017). Within the range of 10–50 μg/mL, the scavenging rates of *Lycium barbarum* pigments on DPPH, ABTS, and OH radicals can reach 37.91%, 65.49%, and 82.64%, respectively, and the ability to scavenge radicals increases with increasing concentrations of *Lycium barbarum* pigments (Guo 2023). The total flavonoids and phenols of LF exhibited good scavenging ability against DPPH and ABTS free radicals, and the total flavonoid content was significantly positively correlated with the ABTS free radical scavenging rate, whereas the total phenol content was significantly positively correlated with the ABTS and DPPH free radical scavenging rates (Lu, Mi, et al. 2019).

In vitro antioxidant assessments revealed that the LF water extract can increase the levels of SOD and GSH in ARPE cells, reduce the content of reactive oxygen species (ROS), promote Nrf2 entry into the nucleus, increase hemoglobin levels, significantly improve cell viability, and alleviate oxidative stress damage (Zheng, Li, et al. 2023). LF extract can increase the activity of SOD and CAT in *Caenorhabditis elegans* in a dose‐dependent manner, thereby enhancing its resistance to oxidative stress (Xiong 2021). LF polysaccharides can increase the activities of SOD, CAT, and TC in the cerebral cortex of D‐galactose‐induced aging mice, effectively improving the redox status of the mouse cerebral cortex and thereby improving hippocampal function in aging mice and enhancing motor, spatial exploration, learning, and memory abilities (Liu, Li, et al. 2020). It can also exert a protective effect on aging mice by increasing the activities of SOD, CAT, and GSH‐Px in the serum and reducing MDA levels (Yi et al. 2013). Fatigue spinning stick, running, and weight‐bearing swimming experiments demonstrated that LF polysaccharides could significantly prolong the exercise time of mice, improve exercise ability, and alleviate fatigue (Liu, Jin, et al. 2016). Its mechanism involves increasing the activity of SOD and GSH‐Px in the serum, liver, and muscle, reducing the content of MDA and ROS in the liver and muscle, and increasing oxidative stress.

#### 3.2.2 *Lycium barbarum* Leaves and Extracts

In vitro studies demonstrated that LL flavonoids possess significant scavenging abilities against O^2−^, OH^−^, DPPH^−^, NO^−^, and ABTS free radicals, with maximum scavenging rates of 62%, 84%, 80%, 34%, and 78%, respectively. When the concentration was 0.5 mg/mL, the ability of LL flavonoids to reduce Fe^3+^ reached 50% of that of Vc and BHT (Fan et al. 2017). The LL polysaccharides obtained from different concentrations of ethanol fractionation (30%, 50%, and 70%) possess a strong ability to scavenge ABTS^+^, DPPH^+^, OH, and O^2−^ free radicals, of which the LL polysaccharides obtained from 30% and 50% ethanol fractionation exhibited the strongest ability to scavenge radicals (Wei et al. 2018). Comprehensive studies have demonstrated that polyphenols, alkaloids, polysaccharides, and proteins in LL possess certain scavenging abilities against ABTS, DPPH, OH, and O^2−^ free radicals, and exhibit a clear dose–response relationship with mass concentration. In terms of antioxidant capacity, polyphenols from LL possess the best antioxidant capacity, followed by alkaloids and polysaccharides, whereas LL has the weakest antioxidant capacity (Ma 2024b). Zhao (2020) reported that the water extract, ethanol extract, and polysaccharides from LL all possess certain scavenging abilities against ABTS and DPPH radicals. Among them, the water extract of LL exhibited strong DPPH scavenging activity, with an IC_50_ value of 0.017 ± 0.002 mg/mL; the ethanol extract of LL possessed strong ABTS scavenging activity, with an IC_50_ value of 0.351 ± 0.025 mg/mL. The Fe^3^ + −reduction ability experiment demonstrated that the water extract of LL had the highest antioxidant activity, followed by the LL polysaccharides. The ethanol extract of LL had the weakest effect.

In vivo studies confirmed the antioxidant effects of LL and its extracts. LL flavonoids can upregulate the mRNA expression of the antioxidant enzymes sod‐2, gcs‐1, and skn‐1 through the MAPK pathway, thereby prolonging the lifespan of *Caenorhabditis elegans* under oxidative stress (Niu et al. 2022). It can effectively increase SOD activity in mouse serum and brain tissue, reduce MDA levels, and exert a protective effect on depressed mice by clearing free radicals and enhancing antioxidant capacity (Wang, Yue, et al. 2015). LL flavonoids also exert significant antioxidant effects on n‐linolenic, arachidonic, linoleic, lauric, pentadecanoic, and myristic acids, thus exhibiting good antifatty acid oxidation effects in lamb mince (Chen et al. 2018). Water extracts of LL increase the activities of SOD and GSH in the serum of aging model mice, reduce MDA levels, and increase the expression of the antioxidant proteins Nrf2 and HO‐1, thereby improving learning and memory (Tong et al. 2024). LL polysaccharides can reduce serum MDA levels in normal mice and increase GSH‐Px and total T‐SOD activity (Bai, Zhang, et al. 2023). Zhou et al. (2023) reported that the antioxidant activity of LL is significantly and positively correlated with the contents of cryptochlorogenic acid, 5‐*O*‐feruloylquinic acid, quercetin‐3‐*O*‐rutinoside‐7‐*O*‐glucoside, chlorogenic acid, and betaine.

#### 3.2.3 *Lycium barbarum* Root Bark and Extracts

Research has demonstrated that with increasing growth duration, the scavenging capacity of DPPH and ABTS radicals, as well as the total reducing ability of LB, decreases. Specifically, the total antioxidant capacity of 1‐year‐old LB was the strongest, whereas the total antioxidant capacities of 3–5‐year‐old LB were similar. The total antioxidant capacity of 7‐year‐old LB was the weakest (Li 2023). Hadjipavlou‐Litina et al. (2009) reported that kukoanin A can interact strongly with DPPH, exhibiting a reduction activity of up to 96% and an excellent radical scavenging ability. Li, Lin, et al. (2018) reported that both kukoanins A and B have good scavenging abilities against DPPH, OH, and O^2−^ radicals. The increased expression of α‐synuclein leads to oxidative stress within the intracellular environment, thereby exacerbating apoptosis. Kukoanin A can reduce the levels of ROS and MDA in PC12 cells, a Parkinson's disease model induced by fisetin, inhibit the expression of the α‐synuclein protein, and increase SOD activity, thereby enhancing cell survival rate (Liu, Song, et al. 2020). The IC_50_ values of the LB polyphenols for their DPPH and ABTS radical scavenging abilities were 20.00 mg/L and 59.37 mg/L, respectively. Its ability to scavenge ROS was greater than that of Trolox at the same concentration. It significantly increased the GSH content and SOD activity in HSF cells damaged by H_2_O_2_ (Zang et al. 2021).

In vivo studies further corroborated the antioxidant effects of LB and its extracts. Kukoanin B can activate the Nrf2/kelch‐like epichlorohydrin‐related protein (Keap)1/antioxidant response element (ARE) signaling pathway, reduce the expression of the oxidative stress‐related protein MDA5 in brain tissue, increase the expression of the antioxidant stress proteins CAT and SOD‐1, and improve the oxidative stress response in rats with traumatic brain injury (Chen and Wang 2022). Kukoanin B can also reduce the mRNA expression levels of SOD1, SOD2, MDA, Nrf2, Keap1, and ARE in lung tissue by regulating the Nrf2/Keap1/ARE pathway, thereby effectively alleviating oxidative stress damage in mice with low‐temperature blast injury (Sun et al. 2022). The ethanol extract of LB reduced the damage caused by oxidative stress in diabetic rats by increasing the activity of SOD and NO in the serum, decreasing the content of MDA, and reducing the expression of iNOS mRNA in the aorta, while increasing the expression of eNOS mRNA (Peng 2014). Recent studies have demonstrated that the total flavonoids and phenolic amide components of LB can scavenge DPPH. Simultaneously, the phenolic amide components of LB scavenge NBT superoxide radicals. LB decoction can also delay skin aging by inhibiting the generation of oxygen‐free radicals in the body (Xu et al. 2021).

### 3.3 Anti‐Inflammatory Effects of *Lycium barbarum*

**FIGURE 7:**
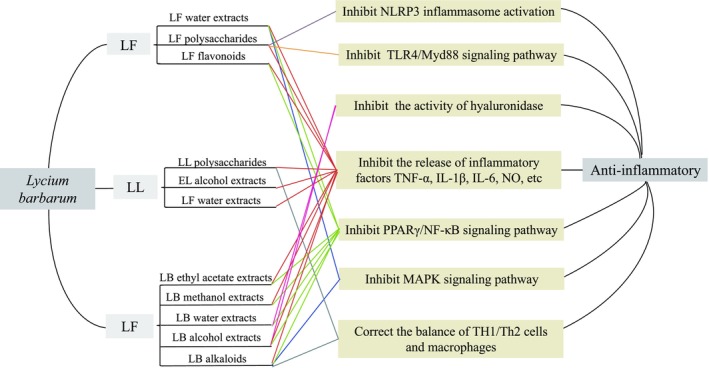
Anti‐inflammatory activity of *Lycium barbarum* in LF, LL, and LB.

*Lycium barbarum* and its extracts can reduce the release of inflammatory factors, such as IL‐1β, IL‐6, IL‐12, and tumor necrosis factor (TNF‐α), by inhibiting signaling pathways, such as the myeloid differentiation factor 88 (MyD88), PPARγ, MAPK, toll‐like receptor 4 (TLR4)/nuclear factor κB (NF‐κB), c‐Jun N‐terminal kinase (JNK), and nod‐like receptor pyrin containing three inflammasome (NLRP3) pathways, thereby alleviating the inflammatory response (Figure 7).

#### 3.3.1 *Lycium barbarum* Fruit and Its Extracts

The water extract of LF can inhibit the secretion of the LPS‐stimulated proinflammatory mediators NO, prostaglandin E_2_ (PGE_2_), TNF‐α, and IL‐6 in RAW 264.7 cells by blocking the MAPK and NF‐κB pathways and suppressing the expression of cyclooxygenase‐2 (COX‐2) and iNOS mRNAs (Oh et al. 2012). LBPs‐1, LBPs‐2, and LBPs‐3, polysaccharides from LF with different molecular weights, can all inhibit the increase in TNF‐α and IL‐6 secretion in BV2 cells induced by LPS, and this activity is closely related to the molecular weight of LF polysaccharides (Hao et al. 2024). LF polysaccharides exert a protective effect on human retinal pigment epithelial cells stimulated with high glucose levels. Its molecular mechanism is related to the inhibition of NLRP3 inflammasome activation, downregulation of GSDMD to reduce apoptosis, and inhibition of the release of the inflammatory factors IL‐1β and IL‐18 (Liang et al. 2023). LF polysaccharides can also reduce the levels of TNF‐α and IL‐6 in the serum of diabetic nephropathy rats and increase the level of IL‐10 by inhibiting the TLR4/Myd88 signaling pathway, thereby reducing the degree of vacuolar degeneration and inflammatory infiltration of glomerular epithelial cells (Qu, Bian, et al. 2020). LF flavonoids can promote PPARγ activation at the protein and mRNA levels in the macrophages and colon tissues of mice with colitis, inhibit NF‐κB and p65 expression and nuclear translocation, suppress the secretion of the inflammatory factors IL‐1β, TNF‐α, and IL‐6 in macrophages and colitis in the colon and serum, improve inflammatory infiltration induced by dextran sulfate sodium salt (DSS), and restore the colon mucosal structure; this anti‐inflammatory mechanism is related to targeted regulation of the PPARγ/NF‐κB pathway (Deng 2023).

#### 3.3.2 *Lycium barbarum* Leaves and Extracts

An imbalance in the Th1/Th2 cell ratio is a key factor in the development of asthma. LL polysaccharides can reduce the infiltration of inflammatory cells in the nasal mucosa, increase the proportion of Th1 cells in the spleen, decrease the proportion of Th2 cells, correct the imbalance in Th1/Th2 cell ratios, and exert a protective effect in mice with ovalbumin (OVA)‐induced allergic rhinitis (Zhao, Guo, et al. 2020; Zhao, Xiao, et al. 2020). LL polysaccharides can reduce the levels of TNF‐α, IL‐4, IL‐6, monocyte chemoattractant protein‐1 (MCP‐1), and IL‐17A in the plasma and bronchoalveolar lavage fluid of asthmatic mice (Cui et al. 2020), decrease the infiltration of eosinophils in lung tissue, alleviate pulmonary fibrosis, promote macrophage differentiation toward M1, increase the proportion of Th1 cells and the number of M1 macrophages in lung tissue, reduce the number of M2 cells, and inhibit airway inflammation in allergic asthmatic mice (Bai, Wu, et al. 2023). The water extract and 80% alcohol precipitation supernatant of LL can downregulate the expression of the proinflammatory factors TNF‐α, IL‐1β, and IFN‐γ in the hippocampus of subacute aging mice and upregulate the expression of the anti‐inflammatory factor IL‐10, thereby reducing neuronal apoptosis caused by inflammatory stress and exerting neuroprotective effects (Tong et al. 2024).

#### 3.3.3 *Lycium barbarum* Root Bark and Its Extracts

The ethyl acetate extract and the methanol extract of LB suppressed the TNF‐α‐induced activation of NF‐κB, whereas the dichloromethane extract activated PPARγ. Further research demonstrated that phenolic amides (N‐trans‐feruloyltyramine, N‐trans‐caffeoyltyramine, N‐trans‐p‐hydroxycinnamoyl tyramine, and dihydrocaffeoyltyramine) were the major components from LB responsible for NF‐κB inhibition, with N‐trans‐caffeoyltyramine exhibiting a stronger inhibitory effect on NF‐κB (IC_50_ = 18.4 μmol/L). Fatty acids were identified as the major plant constituents responsible for PPARγ activation (Xie et al. 2014). N‐trans‐feruloyltyramine, the active ingredient of LB, can inhibit the production of NO and PGE2 in RAW 264.7 macrophages induced by LPS by regulating the activator protein 1, MAPK, and JNK signaling pathways, thereby exerting anti‐inflammatory effects (Jiang et al. 2015). The water extract of LB can upregulate the expression of PPARγ in adipocytes after LPS intervention, thereby alleviating the LPS‐induced inflammatory response and improving glucose metabolism in these cells (Yu and Ye 2012). HA degradation products can promote an inflammatory response during wound healing. The water and ethanol extracts of LB inhibited the activity of hyaluronidase, with the water extract exhibiting the highest inhibition rate, indicating that LB can inhibit the skin inflammatory response (Li et al. 2011). The ethanol extract of LB can inhibit the activation of NF‐κB in the renal tissue of diabetic nephropathy rats and reduce the levels of serum TNF‐α and IL‐6, thereby reducing the renal pathological damage caused by inflammatory reactions and improving renal function (Yang and Ye 2008). Kukoamine A can reduce the levels of TNF‐α, IL‐6, and IFN‐γ in the serum and IL‐4 in the middle ear lavage fluid of rats with secretory otitis media induced by OVA in a dose‐dependent manner and can inhibit inflammation‐mediated ear mucosal swelling (Kadiliya et al. 2020).

### 3.4 Immunomodulatory Effects of *Lycium barbarum*

Immunomodulation is the process by which the immune system adjusts or balances immune responses to different stimuli and demands. The primary function of the immune system is to recognize and resist invading pathogens, and an excessive or decreased immune response can lead to disease (Wu et al. 2024). *Lycium barbarum* and its extracts can regulate immune function by increasing the weight of immune organs, activating macrophages, promoting T‐cell immune responses, and increasing the number of B cells.

#### 3.4.1 *Lycium barbarum* Fruit and Its Extracts

LB polysaccharides can improve peripheral blood lymphocyte counts, promote cell cycle recovery in bone marrow cells, restore the cytotoxicity of natural killer cells, alleviate spleen lesions, and reverse the immune toxicity caused by doxorubicin (Dox) in mice (Deng et al. 2018). It can also increase the thymus index, spleen index, IL‐2 and TNF‐α levels, CD3 + T and CD4 + T‐cell percentages, and CD4 + T/CD8 + T values in liver cancer model mice, effectively reducing the immunosuppressive effect of cisplatin (Wang et al. 2018). LB polysaccharides can also protect immune organs, increase the production of immune‐related cytokines, and prevent hepatotoxicity in cyclophosphamide (CTX)‐induced mice (Ding et al. 2019). The water‐soluble polysaccharides LBPC2, LBPC4, LBPB1, and LBPA3 from LB can increase the weight of immune organs (spleen and thymus) in normal mice and reduce CCL4‐induced liver toxicity. Their activities are in the order of LBPC2 > LBPC4 > LBPB1 > LBPA3 (Li et al. 2003). Luo et al. (2022) reported that LB powder and LB polysaccharides can restore body weight and feed intake; improve spleen, thymus, and colon tissue damage in mice; effectively promote the secretion of TNF‐α, IL‐1β, IFN‐γ, and immunoglobulin A (IgA) in immunosuppressed mice; increase the expression of the immune‐related genes TNF‐α, IL‐1β, and IFN‐γ in the colon; and reverse the immunosuppression induced by CTX. LB decoction can increase the initial T‐cell generation and output function of the thymus in D‐galactose‐induced immune aging mice (Zhang and Liang 2008); increase the spleen index, thymus index, lymphocyte transformation rate, and hemoglobin released by red blood cells in normal mice; and promote humoral and cellular immune function (Wang et al. 2006). Notably, the residue of LB juice still contains LB polysaccharides (3.64% ± 0.14%) and vitamin C (7.14% ± 0.86%), which can increase the liver, thymus, and spleen indices of normal mice; increase the macrophage phagocytic index and percentage; increase the E‐rose ring formation rate; and enhance the immune function of mice (Yan et al. 2012).

#### 3.4.2 *Lycium barbarum* Leaves and Extracts

The total flavonoids, polysaccharides, vitamin C, taurine, and betaine from LL can increase the liver, thymus, and spleen indices of normal mice in a dose‐dependent manner; increase the macrophage phagocytic index and percentage; increase the E‐rose ring formation rate; and enhance the immune function of mice (Yan et al. 2012). LL decoction can significantly increase the weight of the thymus and spleen in normal mice, improve the percentage of ANAE cells and the T lymphocyte transformation rate, and enhance humoral and cellular immune functions (Jia et al. 2014). LL flavonoids can significantly improve the thymus and bursa indices of broiler chickens, increase the serum levels of IgG and IgA, and improve the immune performance of broiler chickens after transportation stress (Wu 2022). Flow cytometry analysis revealed that polysaccharides from LL can increase the number of T, B, and dendritic cells (DCs) in the spleens of normal mice, thereby enhancing the immune system (Bai, Zhang, et al. 2023). Helper T (Th) cells, a subset of T lymphocytes, play a crucial role in immune regulation, and an imbalance between Th1/Th2 cells has been reported in various allergic and autoimmune diseases. LL polysaccharides can increase the proportion of Th1 (CD4+ and IFN‐γ+) cells in the spleen, reduce the proportion of Th2 (CD4+ and IL‐4+) cells, and correct the imbalance of Th cells (Zhao, Guo, et al. 2020; Zhao, Xiao, et al. 2020).

#### 3.4.3 *Lycium barbarum* Root Bark and Extracts

After binding to suppress tumorigenicity 2, IL‐33 induces the migration of numerous Th2 cells to the site of inflammation, leading to a series of immune‐inflammatory reactions dominated by Th2 cells. Kukoamine A can inhibit the expression of the IL‐33 and ST2 proteins in the middle ear mucosal tissue of rats with otitis media, increase the levels of the Th1 cytokine IFN‐γ in ear vesicle lavage fluid and serum, reduce the level of the Th2 cytokine IL‐4, and correct the Th1/Th2 cell immune imbalance (Liao et al. 2023). LB extracts can interact with the immune checkpoint programmed death ligand 1 (PD‐L1), block its binding to programmed death receptor‐1 (PD‐1), restore immune cell function, promote T‐cell killing of tumor cells, regulate mouse immune function, and exert anticancer effects (Yin 2021). LB polysaccharides can reverse leukopenia caused by cyclophosphamide and coirradiation but have no significant effect on increasing the weight of immune organs (Wang et al. 1993). Cinnamic acid, the active ingredient of LB, increases white blood cell counts and reverses leukopenia and thrombocytopenia caused by radiation‐induced damage (Cao and Wang 2002). Xiong and Li (1991) reported that LB had different regulatory effects on different immune states. The decoction inhibited the secretion of IL‐2 by normal mouse spleen cells, significantly enhanced the decrease in IL‐2 secretion by cyclophosphamide‐induced mouse spleen cells, and strongly inhibited the increase in IL‐2 secretion induced by azathioprine.

### 3.5 Anti‐Osteoporosis Effects of *Lycium barbarum*

Osteoporosis (OP) is a chronic bone metabolic disease that makes bones prone to fracture due to the weakness caused by a reduction in bone density and destruction of the bone tissue microstructure. It is associated with a high incidence and mortality rate (Amin et al. 2023). *Lycium barbarum* and its extracts primarily exert anti‐osteoporotic effects by regulating cytokines, increasing estrogen levels, and enhancing bone density, osteogenic activity, and mineralization ability.

#### 3.5.1 *Lycium barbarum* Fruit and Its Extracts

Bone mineral density (BMD) is the gold standard for the clinical diagnosis of OP. The water extract of LF can exert a protective effect on ovariectomized rats by increasing the levels of BMD, TGF‐β1, NO, NO synthase, phosphate ions, and osteocalcin, and reducing the levels of calcium ions, magnesium ions, ALP, IL‐6, and TNF‐α in the serum. This protective effect was positively correlated with the concentration of LF extract (Hou and Sun 2019). The ethyl acetate extract of LF increased the femoral BMD, bone volume ratio, trabecular thickness, and trabecular number in rats with ovariectomy‐induced OP while reducing the trabecular spacing and bone surface area ratio (Liu, Yang, et al. 2016). Zheng, Hua, et al. (2023) reported that the water extract of LF after alcohol extraction, LF polysaccharides, and elution solutions of 30% ethanol, 60% ethanol, and 95% ethanol after dissolving the LF alcohol extract in water improved the formation area of the first vertebra, staining optical density value, and number of vertebrae and joints in zebrafish to varying degrees, with goji berry polysaccharides exhibiting the best effect. LF polysaccharides significantly inhibited the formation of bone resorption cavities in zebrafish scales, enhanced ALP activity in scales, reduced tartrate‐resistant acid phosphatase (TRAP) activity, upregulated the expression of the bone formation‐related genes alp, sp7, and opn, and downregulated the expression of the bone resorption‐related genes ctsk and tracp, thereby regulating the dynamic balance between bone formation and resorption and exerting anti‐OP effects (Zheng, Hua, et al. 2023).

#### 3.5.2 *Lycium barbarum* Leaves and Extracts

LL water extract could increase the expression of estrogen receptor (ER) α and β in the lumbar spine and the serum E_2_ levels of postmenopausal OP rats and improve the BMD in the vertebrae, femur, and tibia (Ma et al. 2016). Wang, Ren, et al. (2015) reported that intervention with LL can reverse the decrease in BMD of the vertebrae, femurs, and tibias caused by ovariectomy in rats, thereby delaying bone loss. Although research investigating the effects of LL and its extracts in OP is limited, existing studies have demonstrated that kaempferol (Pan, Zhang, et al. 2024), quercetin (Li, Zhang, et al. 2018; Taskan and Gevrek 2020), and other compounds can increase BMD, improve bone microvascular circulation, enhance bone microstructure, inhibit osteoclast activation, stimulate osteoblast activity, maintain the dynamic balance and bone homeostasis of osteoblasts/osteoclasts, and inhibit bone resorption by intervening in signaling pathways such as the Wnt/β‐catenin, RANKL/RANK/OPG, PI3K/Akt, ER/ERK, MAPK, and BMP/samd pathways.

#### 3.5.3 *Lycium barbarum* Root Bark and Extracts

Kim et al. (2016) reported that the water extract of LB not only inhibits RANKL‐induced osteoclast differentiation by reducing the synthesis and secretion of osteoclastogenesis markers (RANK and TRAP) but also reduces the expression of the osteoclastogenesis‐related markers NFATc1, c‐Fos, and CAII and reduces bone density loss and trabecular area loss in an OVX rat model. The combination of LB extract and *Achyranthes japonica* effectively mineralizes bone formation and promotes osteoblast differentiation via the upregulation of bone metabolic markers (Runx2, Alpl, and Bglap), prevents OVX‐induced BMD loss, and improves trabecular bone structural properties in an OP mouse model by inhibiting osteoclastogenesis (Park et al. 2019). The ethanolic extract of LB promoted the proliferation and differentiation of C3H10T1/2 and MC3T3‐E1 osteoblasts; increased ALP activity; upregulated the expression of osteoblast‐inducing genes, such as Alpl, Runx2, and Bglap; and prevented OVX‐induced BMD loss in mice by promoting the differentiation of osteoblast lineage cells (Paek et al. 2014). Yin et al. (2001) reported that water extracts and 30%, 60%, and 90% ethanol extracts of LB promoted the proliferation of UMR106 osteoblast‐like cells and inhibited the proliferation of osteoclasts.

### 3.6 Antitumor Effects of *Lycium barbarum*

Cancer has a high incidence rate and mortality rate and is a major public health problem that crucially threatens human health. With the advancements in scientific research, the antitumor effects of *Lycium barbarum* and its extracts have gradually become a research hotspot, and *Lycium barbarum* has the potential to yield a new generation of antitumor drugs. Currently, research examining the antitumor mechanism of *Lycium barbarum* and its extracts has focused on four key aspects: regulating the cell cycle, modulating immune function, inhibiting tumor cell proliferation, invasion, and migration, and regulating the expression of related proteins and genes, such as those involved in apoptosis.

#### 3.6.1 *Lycium barbarum* Fruit and Its Extracts

Polysaccharides, flavonoids, saponins, and other LF components exhibit good antitumor activity. LF polysaccharides are the most important and well‐studied antitumor components. They can block tumor cells in the G0/G1, S, and G2/M phases by regulating the MAP3K3, NF‐κB, MMTV, PyMT, PD1/PD‐L1, and P53 signaling pathways; induce cancer apoptosis; inhibit tumor cell proliferation, tumor interstitial blood vessel formation, invasion, and metastasis; improve immune escape; reduce chemical drug resistance; improve treatment sensitivity; and play an inhibitory role in digestive and reproductive system tumors (Wang et al. 2018; Jiang and Li 2022; Shan et al. 2010). LF flavonoids can inhibit the proliferation of the gastric cancer cell line SGC‐7901 in a time‐ and dose‐dependent manner, block cells in the G0/G1 phase, and induce apoptosis (Zhang et al. 2014). LF saponins inhibit the proliferation, invasion, and metastasis of breast, lung, and nasopharyngeal cancer cells and induce their apoptosis by regulating the interaction between adipocytes and breast cancer cells (He et al. 2019), targeting nonmuscle myosin IIA (Wei et al. 2016), and inhibiting the expression of the Suv39H1 and Janus kinase 2 (JAK2)/signal transducer and activator of transcription 3 (STAT3) signaling pathways (Hu et al. 2021).

#### 3.6.2 *Lycium barbarum* Leaves and Extracts

Currently, research investigating the antitumor effects of LL is limited. Mohammed et al. (2024) reported that flavonoids from LL, which are rich in rutin, quercetin, kaempferol, and luteolin, can induce apoptosis in the human liver cancer cell line HepG2 through the mitochondrial pathway. Lei (2014) similarly reported that total flavonoids from LL can inhibit the proliferation of human liver cancer HepG2 cells, induce chromatin condensation, nuclear fragmentation, and the formation of apoptotic bodies, and increase their apoptosis rate. These effects may be related to an increase in the expression of the proapoptotic proteins Bax and Caspase‐3, as well as the initiation of the mitochondrial apoptosis pathway.

#### 3.6.3 *Lycium barbarum* Root Bark and Extracts

LB extract can inhibit the growth of subcutaneous tumors in Lewis lung cancer‐bearing mice and suppress the EMT of lung cancer cells by regulating the PI3K/AKT pathway, blocking the binding of PD‐L1 and PD‐1, downregulating the expression of the stromal markers N‐cadherin and vimentin, and increasing the expression of the epithelial marker E‐cadherin, thereby reducing the invasion and migration ability of the lung cancer cell lines A549 and H1299, restoring immune cell function, and promoting the killing of tumor cells by T cells (Yin 2021). Glioblastoma (GBM) is a common primary brain tumor in humans, and kukoamine A has been shown to have inhibitory effects on GBM. These effects are associated with the downregulated expression of B‐cell lymphoma‐2 (Bcl‐2), CCAAT/enhancer binding protein β (C/EBPβ), N‐cadherin, and vimentin, as well as increased levels of Bax, Caspase‐3, and E‐cadherin (Wang et al. 2016).

### 3.7 Regulation of the Gut Microbiota by *Lycium barbarum*

The gut microbiota is the largest group of microorganisms in the human body, and it plays a crucial role in the metabolism of nutrients, earning it the nickname “second genome” of humans. Dysbiosis of the gut microbiota can impact the functions of the host's nervous, endocrine, and immune systems, as well as the digestive and absorptive capacity of the gastrointestinal tract, thereby contributing to the development of various diseases. The polysaccharides, oligosaccharides, iridoids, lignans, and flavonoids in *Lycium barbarum* and its extracts have been demonstrated to maintain the balance of glucose and lipid metabolism, inhibit chemical‐induced intestinal dysfunction, and protect intestinal and joint functions by regulating the gut microbiota.

#### 3.7.1 *Lycium barbarum* Fruit and Its Extracts

The compound 2‐*O*‐β‐D‐glucopyranosyl‐L‐ascorbic acid, an ascorbic acid derivative isolated from LF, exhibits a protective effect on inflammatory bowel disease (IBD), which is attributed to its ability to regulate the abundance of the intestinal microbiota and enhance the composition of the bacterial community (Huang et al. 2019). LF polysaccharides can increase the relative abundance of *Bacteroidaceae, Lactobacillaceae, Verrucomicrobiaceae*, and *Prevotellaceae*; reduce the relative abundance of *Epsilonbacteraeota*, *Firmicutes*, and *Proteobacteria*; and reverse the disruption of the gut microbiota induced by cyclophosphamide (Ding et al. 2019; Luo et al. 2022). LF glycopeptides can enhance the composition of the gut microbiota, increase microbial diversity, reduce the abundance of harmful bacteria, increase the number of beneficial bacteria, restore short‐chain fatty acid levels, and thereby inhibit the malignant progression of colitis (Huang et al. 2022). LF oligosaccharides can effectively regulate the diversity of the gut microbiota; increase the abundance of *Lactobacillus, Bacteroidetes, Prevotella*, and *Akkermansia*; and maintain glucose homeostasis (Liu, Zang, et al. 2022). LF polysaccharides can regulate the composition of the gut microbiota by increasing the abundance of *Romboutsia, Lactobacillus, Fecal*, and *Duroc genera* in the intestines of patients with rheumatoid arthritis (RA), increasing the content of SAM, inducing high methylation of RA‐related gene DNA in the intestinal epithelium, inhibiting the expression of RA‐related genes, and thus improving RA (Lai et al. 2022).

#### 3.7.2 *Lycium barbarum* Leaves and Extracts

Sequencing and analysis of the gut microbiota revealed that LL polysaccharides can enhance mouse intestinal function and improve allergic asthma by increasing the abundance of *Lactobacillus* and *Bifidobacterium* and reducing the abundance of *Firmicutes, Actinobacteria, Alistipes*, and *Clostridiale*s (Bai, Zhang, et al. 2023; Cui et al. 2020). LL water extract can reverse gut microbiota dysbiosis by increasing the abundance of beneficial bacteria, such as *Bifidobacterium* and *Muribaculaceae*, and decreasing the abundance of harmful bacteria, including Bilophila and Lachnoclostridium, thereby suppressing the damage caused by dextran sulfate sodium to the intestine (Yu et al. 2023). The Lachnospiraceae NK4A136 group is positively correlated with E_2_ levels and negatively correlated with follicle‐stimulating hormone, luteinizing hormone, and anti‐Mullerian hormone levels. LL not only increases the abundance of the Lachnospiraceae NK4A136 group but also increases the abundance of Bacteroidetes; reduces the abundance of *Firmicutes, Ileibacterium, Romboutsia*, and *Faecalibaculum*; and protects ovarian function by increasing the diversity and richness of the gut microbiota in polycystic ovary syndrome mice (Zhang, Lu, et al. 2024). LL flavonoids can restore gut dysfunction induced by a high‐fat diet by increasing gut bacterial diversity and altering the composition of the gut bacterial community (Liao et al. 2022).

#### 3.7.3 *Lycium barbarum* Root Bark and Extracts

LB water extract can improve the beta diversity of the gut microbiota in hypertensive rats and improve the abundance and ratio of the gut microbiota at the phylum (Firmicutes and Bacteroidetes), class (Erysipelotrichia and Bacteroidia), order (Erysipelotrichales and Bacteroidales), family (Erysipelotrichaceae, Peptostreptococcaceae, and Muriaculaceae), and genus (*Turicibacter*, *Blautia*, and *Romboutsia*) levels (Shan et al. 2024). However, research investigating the effects of LB on the gut microbiota is lacking; hence, further research is needed.

In summary, although the chemical components or crude extracts of LF, LL, and LB all exhibit pharmacological effects, such as antidiabetic, antioxidative, anti‐inflammatory, immune‐regulatory, anti‐osteoporotic, antitumor, and intestinal flora‐regulatory effects, the components that exert these pharmacological effects possess distinct characteristics. Among them, LF is dominated by polysaccharides, while alkaloids dominate LB, and LL exhibits no obvious characteristics, with comparable amounts of polysaccharides, flavonoids, phenols, alkaloids, and other components. However, studies comparing the efficacy of the same type of components among LF, LL, and LB are currently lacking. Future research can focus on this aspect to fully utilize *Lycium barbarum* resources. Therefore, we have preliminarily summarized the pharmacological effects of LF, LL, and LB, aiming to provide theoretical support for subsequent studies comparing their efficacy.

## 4 Food

As an important component of traditional Chinese medicine, *Lycium barbarum* has a long history of consumption in primary production areas. Recently, it has received increasing attention due to its unique homology with medicine and food. As a homolog of medicine and food, *Lycium barbarum* is highly edible due to its fruits, leaves, and root bark, all of which contain nutrients, such as carbohydrates, proteins, fats, amino acids, vitamins, and minerals (Zhang, Dai, et al. 2024).

### 4.1 *Lycium barbarum* Fruit

The carbohydrate content of LF is approximately 50%–75%, the protein content is approximately 9.0%–13.6% (dry basis), and the fat content is approximately 0.7%–5.0% (dry basis) (Shao et al. 2020), indicating extremely high medicinal value. As of September 2023, data retrieved from the National Special Food Information Query Platform indicated that 1671 health foods contained LF and its extracts. The number of products that use LF as a compatible ingredient was 239, and the number of LF extracts was 1432. Dried LF is the most common form of LF, with 80% of LF sold on the market as dried fruits. In recent years, Ningxia LF has been introduced as a “superfood” from China to Europe, the Americas, and Australia, gaining popularity due to its rich nutritional content and numerous health benefits. Individuals directly consume LF or add concentrated juices or LF extracts to their beverages (Yu et al. 2023).

Fresh LF is a small berry rich in juice that can be easily processed into beverage products. Currently, beverage products include fresh LF juice drinks, fermented drinks, tea drinks, and solid drinks. LF purees effectively preserve the nutritional components of LF. It can be used not only as a terminal product but also as an intermediate raw material to be processed into juice or added to modern tea drinks, representing a novel approach to deep processing of LF, and it has considerable market potential (Zhang, Zhou, et al. 2022). For example, LF juice is a fruit and vegetable juice primarily made from LF puree that can better maintain the color, aroma, and taste of LF. Young consumers appreciate its ready‐to‐drink and portable nature, making it the primary product in the new LF market (Zhang, Zhu, et al. 2024). Fermented LF drinks include soft drinks, fruit wines, vinegar, and yogurt. Fermentation can increase flavor substances, such as 2‐nonanone, while ensuring nutritional value and enhancing taste (Wang, Shan, et al. 2020). LF wine is a low‐alcohol beverage fermented from LF that is low in alcohol and rich in nutritional components, and it has become a representative northern fruit wine (Sun et al. 2020). Li et al. (2021) used SPME‐GC–MS to analyze the flavor compounds of LF juice and wine and reported that the volatile aroma compounds of LF juice were primarily aldehydes and ketones, whereas the volatile aroma compounds of fermented LF wine were primarily esters and alcohols. LF yogurt is a beverage made primarily from LF and fresh milk and is pasteurized and fermented with lactic acid bacteria. Luo et al. (2024) used headspace solid‐phase microextraction (HS‐SPME) technology to detect volatile components in sterilized milk, plain yogurt, LF pure yogurt, and LF yogurt.

The results revealed that the types and contents of volatile flavor compounds were highest in LF yogurt, with an increase in aldehydes, ketones, acids, esters, and terpenes, all of which positively contributed to the formation of the LF yogurt flavor. Tea is a common beverage with a long history in China. LF tea is a composite tea beverage made by mixing LF with tea leaves that are rich in polysaccharides and phenolic amides. LF tea has several health benefits and is commonly available on the market. Currently, 50 types of LF teas are approved by the National Medical Products Administration as health foods (Hu et al. 2021). LF is not only used in beverage products and dried LF, but can also be made into baked goods, jelly, jam, and other products. However, they are currently mostly in the experimental stage, and no related products with high market value are available.

### 4.2 *Lycium barbarum* Leaf

LL has a long history of being edible. As early as the Taiping Shenghui Prescription, the “cure Wulao and Qishang, LL Congee prescription” was recorded. Currently, the developed products are primarily edible, including LL vegetables, LL tea, and others that are applied in Türkiye, Argentina, Palestine, South Africa, Botswana, Somalia, Israel, and other locations (Huang et al. 2019). Adding LL homogenate to the feed improved the growth performance of the rats, increased heart and kidney weights, and significantly reduced the Shannon index of the rat cecum at a dosage of 2 g/kg. Additionally, LL can increase the relative abundance of beneficial bacteria (Guo et al. 2024), suggesting that it can regulate antioxidant and immune functions, thereby positively promoting animal growth and development. Spreading is beneficial for the accumulation of water extracts, amino acids, sugars, and fatty acids in LL tea and can promote the accumulation of pleasant aromatic substances such as dihydroactinidiolide. Moreover, LL tea processed with a moisture content of 55% is characterized by a floral and fruity aroma, a sweet aroma, and a sweet and mellow taste (Zhu et al. 2022; Pan, Duan, et al. 2024). Li, Li, et al. (2024) reported through analyzing the aroma compounds in LL tea that 3,5‐octadien‐2‐one, 2,4‐di‐tert‐butylphenol, methylheptenone, cis‐2‐penten‐1‐ol, pinocarveol, 2,4‐dimethylbenzaldehyde, styrene, benzaldehyde, and n‐hexanal are the characteristic volatile components of the LL tea aroma.

### 4.3 *Lycium barbarum* Root Bark

LB contains various nutrients, including amino acids, trace elements, and vitamins, which provide it with a wide range of health benefits and nutritional value. Currently, LB is predominantly used to assist in the development of hypoglycemic health foods, and its products include dew, teas, and capsules (Lu, Guo, et al. 2019). LB, made primarily from LB, cools blood and relieves muscle heat. Healthy tea drinks composed of LB, mistletoe, wujiapi, and other ingredients can enhance the immune system of individuals with suboptimal health, improving their susceptibility to colds, sore eyes, poor sleep quality, spleen deficiency, and fatigue (Qingdao Hengbo Instrument Co. Ltd. 2017). LB contains guanidine derivatives, and healthy tea drinks composed of mistletoe ( *Cornus officinalis* ) and other components have been shown to reduce blood sugar and cholesterol levels (Qingdao Hengbo Instrument Co. Ltd. 2014). LB health food includes Huangqi Digupi capsules, Ge Gen Digupi capsules, and others, all of which exhibit the health function of assisting in lowering blood sugar (https://www.samr.gov.cn/).

## 5 Safety

According to the Chinese Pharmacopeia, the recommended clinical dose of LF is 6–12 g/day. Furthermore, a maximum tolerance test for LF demonstrated the absence of acute toxicity within a dosage range of 45 g/kg body weight (Qu, Pan, et al. 2020). A toxicity experiment examining LF polysaccharides demonstrated the absence of acute oral toxicity and genotoxicity within a dosage range of 250–10,000 mg/kg body weight (Mu et al. 2002; Zhao et al. 2015). The nutritional value of LF juice drinks, pure LF, and LF sauce was confirmed. Safety evaluations conducted in mice revealed no signs of acute or chronic oral toxicity. Additionally, genetic toxicity tests, including the Ames test, bone marrow cell micronucleus test, and sperm malformation test, indicated the absence of mutagenicity (Chen et al. 2013; Qin et al. 1988). Furthermore, a 30‐day feeding trial (body medicine‐taking experiment) focused on LF juice drinks revealed no significant toxic reactions in any of the measured indices, such as routine blood tests, routine urine tests, liver and kidney function tests, electrocardiogram examinations, and improvements in symptoms, such as eye swelling, eye pain, photophobia, blurred vision, and dry eyes, in subjects with visual fatigue (Chen et al. 2013). Toxicological safety experiments conducted on compound LL tea demonstrated no significant adverse effects on growth and development, weight, activity level, or mental state in rats fed a dose equivalent to 200 times the recommended dose for 7 days (Ma et al. 2000). The administration of LL flavonoids did not induce acute or subchronic toxicity in mice (Mei 2016). The treatment of rabbits with 80 g/kg gavage or 60 g/kg intraperitoneal injection of LB resulted in no adverse effects (Pharmacology Teaching and Research Group 1959). In summary, LF, LL, and LB do not exhibit acute, chronic, or genotoxic toxicity and can be safely incorporated into a healthy diet.

## 6 Conclusion and Perspectives

**FIGURE 8:**
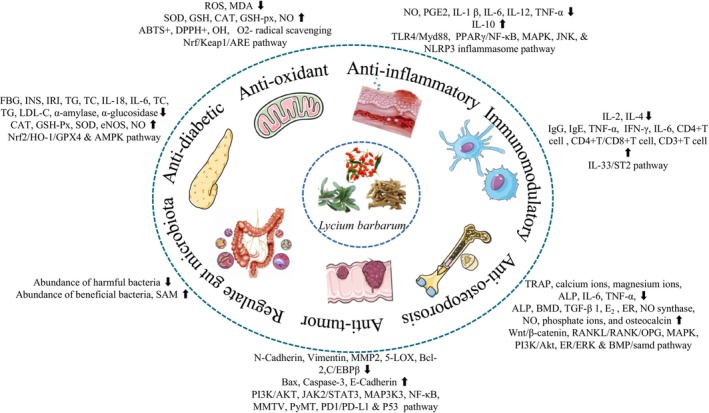
Pharmacological activity and mechanism/targets of *Lycium barbarum*.

*Lycium barbarum* has a high yield and nutritional value. As a medicinal and edible food as well as a “superfood,” *Lycium barbarum* has great research and development prospects both domestically and internationally. Numerous studies have revealed that *Lycium barbarum* exhibits various health benefits (Figure 8), including lowering blood sugar levels, promoting antioxidant, anti‐inflammatory, and anti‐osteoporotic activities, regulating immune function and antitumor effects, and modulating the gut microbiota. The benefits of *Lycium barbarum* primarily rely on its abundant bioactive compounds, including flavonoids, polysaccharides, alkaloids, polyphenols, amino acids, and trace elements, as well as its nutritional value.

In addition to current large‐scale applications in Chinese medicine, *Lycium barbarum* also has broad development prospects in several aspects. First, functional foods and nutritional supplements, such as polysaccharides, pigments, and flavonoids from LF; flavonoids, polysaccharides, and polyphenols from LL; and polyphenols, flavonoids, and alkaloids from LB, all have good antioxidant and free radical scavenging abilities and can be developed into antioxidant supplements to reduce oxidative stress linked to aging, cardiovascular disease, and neurodegenerative disorders. The polysaccharides in LF, LL, and LB have been well documented to enhance immune function by stimulating the activity of macrophages, T cells, and natural killer cells, making them suitable for functional foods that target immune support. The carotenoids in wolfberry fruits, especially zeaxanthin, can be selectively deposited in the macular region of the retina, protecting the retina from oxidative damage and preventing age‐related macular degeneration and cataracts (Zheng, Hua, et al. 2023). This property can be leveraged to develop nutritional supplements for eye health protection. Next, in the field of cosmetics, LF, LL, and LB are rich in various antioxidant components, and their extracts can be developed into antiwrinkle sera, antiaging creams, firming masks, and other products; LF, LL, and LB, as well as their extracts, have anti‐inflammatory, antioxidant, and antibacterial effects, which can prevent dental caries, gingivitis, oral ulcers, and other oral problems; improve oral health; and have the potential to be developed into toothpaste, mouthwash, and chewing gum. Finally, owing to the effects of LF, LL, and LB in regulating flora and enhancing immune function, they can be developed into poultry feed, livestock feed, and aquatic feed to improve growth performance, immune function, and meat quality. Additionally, their bacteriostatic and insecticidal effects can be utilized to develop biological pesticides. However, to achieve the comprehensive development and utilization of *Lycium barbarum* , the following issues still need to be addressed.

1. Quality control: Treatment of diseases with Chinese herbal medicine primarily relies on the active ingredients it contains, and one of the purposes of quality control is to ensure the effectiveness of Chinese herbal medicine. Phytochemical studies have isolated more than 285 compounds from *Lycium barbarum*. Whether the reported active compounds exist in each *Lycium barbarum* medicinal part (leaf, root bark, and fruit) or their contents in each medicinal part remains unclear. Moreover, the accumulation of various bioactive components varies during the different growth stages. Even at the same growth stage, the types and contents of various bioactive ingredients vary depending on their location and can be influenced by the storage techniques employed. Thus, standardization and uniformity are essential for the development of the *Lycium barbarum* industry.
2. Pharmacology: First, *Lycium barbarum* exerts diverse pharmacological effects, and LF, LL, and LB exert similar pharmacological effects. However, comparative studies investigating the strength of their pharmacological effects that can easily lead to mixing or misuse are lacking. Corresponding experiments must be conducted at the later stages to select the appropriate parts for drug administration. Research has revealed that the total flavonoid, total polysaccharide (Yan et al. 2024), and betaine (Ma 2024a) contents in LL are higher than those in LF or LB. However, research investigating the pharmacological effects of LL is relatively limited, which further limits its medicinal value. In vitro and in vivo pharmacological studies of the functional composition have demonstrated partial traditional uses of *Lycium barbarum* , whereas some traditional uses of *Lycium barbarum* are lacking. The available pharmacological studies are insufficient for assessing ethnobotanical use. Finally, the research object of pharmacological effects is the original medicinal material or extract of *Lycium barbarum* that is mostly present in the form of compound formulas in clinical applications. Research examining the roles, compatibility rules, and related mechanisms of *Lycium barbarum* formulations lacks. In‐depth research must be conducted by combining modern research techniques such as the metabolomics of formulas and syndromes.
3. Research and development of foods: First, the approved registered *Lycium barbarum* health food primarily uses LF and its extracts as raw materials, and research and development of functional and health foods related to LL and LB are lacking. Second, health functions primarily focus on enhancing immunity and relieving physical fatigue and antioxidation. A few products with other health functions exist, and the phenomenon of product homogenization is crucial. Finally, the composition of *Lycium barbarum* mixed with various raw materials is complex, and the stability of liquid formulations is poor. This can result in the formation of precipitates and layers, leading to the predominance of solid formulations in the application of *Lycium barbarum* in health foods. In summary, due to deviations in the utilization of raw materials, limited development of health functions, and single formulation research and development, the target audience for this product is limited, and this restricts the development of *Lycium barbarum* health foods. Therefore, we must delve deeper into high‐value‐added products and advance key technological research to upgrade the industry.
4. Safety evaluation: Although *Lycium barbarum* was recorded as a nontoxic herb in the traditional Chinese medicine system and the safety of products derived from *Lycium barbarum* has been validated, its toxic effects on patients with underlying diseases or in special populations such as the elderly, children, pregnant women, and nursing mothers remain unclear and should be further assessed.

## Author Contributions

Mengfan Peng: wrote original draft, supervision, validation, funding acquisition. Caixia Li: formal analysis, investigation, wrote original draft, software. Xue Yang and Tingting Ye: collect and organize literature. Baosong Liu: methodology, conceptualization, supervision, validation.

## Conflicts of Interest

The authors declare no conflicts of interest.

## Data Availability

Data sharing not applicable to this article as no datasets were generated or analysed during the current study.

## References

[fsn371018-bib-0001] Amin, U. , A. Mcpartland , M. O'sullivan , and C. Silke . 2023. “An Overview of the Management of Osteoporosis in the Aging Female Population.” Women's Health (London, England) 19: 17455057231176655. 10.1177/17455057231176655. PMC10214060 37218715

[fsn371018-bib-0002] Bai, M. , J. Wu , Y. Zhang , W. Zhang , Q. Wang , and J. Zhao . 2023. “ *Lycium Barbarum* Leaf Polysaccharides Regulate T Cells and Macrophages to Relieve Airway Inflammation in Mice With Allergic Asthma.” Journal of Ningxia Medical University 45, no. 5: 456–461+474. 10.16050/j.cnki.issn1674-6309.2023.05.005.

[fsn371018-bib-0003] Bai, M. , Y. Zhang , Q. Li , et al. 2023. “Effect of *Lycium Barbarum* Leaves Polysaccharides on Biological Metabolism, Antioxidant Capacity, Immune Function and Intestinal Microbiota in Mice.” Science and Technology of Food Industry 44, no. 17: 413–419. 10.13386/j.issn1002-0306.2022110055.

[fsn371018-bib-0004] Bondia‐Pons, I. , O. Savolainen , R. Torronen , J. Martinez , K. Poutanen , and K. Hanhineva . 2024. “Metabolic Profiling of Goji Berry Extracts for Discrimination of Geographical Origin by Non‐Targeted Liquid Chromatography Coupled to Quadrupole Time‐Of‐Flight Mass Spectrometry.” Food Research International 63: 132–138. 10.1016/j.foodres.2014.01.067.

[fsn371018-bib-0005] Cai, H. , X. Yang , Q. Cai , B. Ren , H. Qiu , and Z. Yao . 2017. “ *Lycium Barbarum L*. Polysaccharide (LBP) Reduces Glucose Uptake via Down‐Regulation of SGLT‐1 in Caco2 Cell.” Molecules 22, no. 2: 341. 10.3390/molecules22020341. 28241438 PMC6155582

[fsn371018-bib-0006] Cao, J. , and S. Wang . 2002. “Pharmacological Effects and Application of Different Doses of *Cortex Lycii* .” Journal of Clinical and Experimental Medicine 4: 262–263.

[fsn371018-bib-0007] Chen, J. , L. Han , M. Han , and L. Fan . 2018. “Effects of the Flavonoids From *Lycium Barbarum* Leaves on Fatty Acids of Compound Minced Mutton.” China Condiment 43, no. 9: 23–29.

[fsn371018-bib-0008] Chen, L. , and Y. Wang . 2022. “Effect of Kukoamine B on Oxidative Stress Induced by Traumatic Brain Injury in Rats.” Progress of Anatomical Sciences 28, no. 4: 421–423+428. 10.16695/j.cnki.1006-2947.2022.04.012.

[fsn371018-bib-0009] Chen, W. , Y. He , F. Tang , and X. Chen . 2023. “ *Lycium Barbarum* Polysaccharide Improves Endothelium‐Dependent Vascular Relaxation in Diabetic Rats by Down‐Regulating the Calpain‐1 Protein Expression.” Chinese Journal of Integrated Traditional and Western Medicine 43, no. 12: 1486–1490.

[fsn371018-bib-0010] Chen, W. , R. Huang , and D. Liu . 2013. “Safety and Toxicology Evaluation of an Alleviate Eyestrain Wolfberry Beverage.” Modern Food Science and Technology 29, no. 5: 1112–1118+1135. 10.13982/j.mfst.1673-9078.2013.05.016.

[fsn371018-bib-0011] Cui, C. , B. Han , N. Zhang , Z. Zhang , and Y. Li . 2016. “Hypoglycemic Effect of Digupi Water Decoction on Three High Blood Glucose Models in Mice.” China Pharmacist 19, no. 11: 2023–2026.

[fsn371018-bib-0012] Cui, F. , C. L. Shi , X. J. Zhou , et al. 2020. “ *Lycium Barbarum* Polysaccharide Extracted From *Lycium Barbarum* Leaves Ameliorates Asthma in Mice by Reducing Inflammation and Modulating Gut Microbiota.” Journal of Medicinal Food 23, no. 7: 699–710. 10.1089/jmf.2019.4544. 32392444

[fsn371018-bib-0013] Deng, M. 2023. The Mechanism Study Evaluating the Anti‐Colitis of Total Flavonoids of *Lycium Barbarum* . Yanbian University. 10.27439/d.cnki.gybdu.2022.000766.

[fsn371018-bib-0014] Deng, X. , S. Luo , X. Luo , et al. 2018. “Fraction From *Lycium barbarum* Polysaccharides Reduces Immunotoxicity and Enhances Antitumor Activity of Doxorubicin in Mice.” Integrative Cancer Therapies 17, no. 3: 860–866. 10.1177/1534735417753544. 29355051 PMC6142073

[fsn371018-bib-0015] Ding, Y. , Y. Yan , D. Chen , et al. 2019. “Modulating Effects of Polysaccharides From the Fruits of *Lycium barbarum* on the Immune Response and Gut Microbiota in Cyclophosphamide‐Treated Mice.” Food & Function 10, no. 6: 3671–3683. 10.1039/c9fo00638a. 31168539

[fsn371018-bib-0016] Duan, H. , G. Liu , W. Song , and W. Yan . 2024. “Application of Fruits of *Lycium Barharum L*. in Health Food in China.” Science and Technology of Food Industry 45: 12–23. 10.13386/j.issn1002-0306.2023060225.

[fsn371018-bib-0017] Fan, Y. , L. Han , L. Fu , X. Meng , and J. Tian . 2017. “Study on Free Radical Scavenging Effect of Flavonoids From *Lycium Barbarum* Leaves In Vitro.” China Condiment 42, no. 12: 32–37.

[fsn371018-bib-0018] Fang, Z. , F. Liu , J. Fu , and S. Chen . 2004. “Experimental Study on the Hypoglycemic Effect of Cortex Lycii.” Acta Chinese Medicine and Pharmacology 4: 47–48. 10.19664/j.cnki.1002-2392.2004.04.026.

[fsn371018-bib-0019] Gan, X. , J. Wang , Y. Li , and B. Li . 2021. “Lycium Ruthenicum Murray Fruit: Chemical Composition Analysis by Ultra‐High Performance Liquid Chromatography Coupled to Triple Time of Flight Mass Spectrometry and Determination of Total Anthocyanins.” Food Science 42, no. 18: 185–190.

[fsn371018-bib-0020] Gao, Y. , Y. Wei , Y. Wang , F. Gao , and Z. Chen . 2017. “ *Lycium barbarum* : A Traditional Chinese Herb and a Promising Anti‐Aging Agent.” Aging and Disease 8, no. 6: 778–791. 10.14336/AD.2017.0725. 29344416 PMC5758351

[fsn371018-bib-0021] Guo, F. , and X. Dong . 2024. “Study on Hypoglycemic Activity of *Lycium Barbarum* Polysaccharides.” Food Industry 45, no. 3: 136–140.

[fsn371018-bib-0022] Guo, X. 2023. Study on Extraction and Purification of *Lycium barbarum* Pigment and Its Stability and Oxidation Resistance. Tianjin University of Science and Technology. 10.27359/d.cnki.gtqgu.2023.000151.

[fsn371018-bib-0023] Guo, Y. , J. Liu , T. Qiang , M. Wanapat , and G. Xin . 2024. “The Effect of Dietary Supplementation of *Lycium Barbarum* Leaves on the Growth Performance, Organ Indexes and Intestinal Microflora of Rats.” Frontiers in Veterinary Science 11: 1416793. 10.3389/fvets.2024.1416793. 39144075 PMC11322056

[fsn371018-bib-0024] Hadjipavlou‐Litina, D. , T. Garnelis , C. M. Athanassopoulos , and D. Papaioannou . 2009. “Kukoamine A Analogs With Lipoxygenase Inhibitory Activity.” Journal of Enzyme Inhibition and Medicinal Chemistry 24, no. 5: 1188–1193. 10.1080/14756360902779193. 19772491

[fsn371018-bib-0025] Hao, G. , Y. Cao , N. Zhao , J. Liu , and D. Di . 2024. “Separation and Purification of *Lycium Barbarum* Polysaccharides in Neuroinflammation by High‐Speed Countercurrent Chromatography.” Science and Technology of Food Industry 45, no. 20: 1–19. 10.13386/j.issn1002-0306.2023110273.

[fsn371018-bib-0026] Harsh, M. 1989. Tropane Alkaloids From Lycium barbarum linn., In Vivo and In Vitro. Environmental Science, Medicine, Biology, Chemistry Current Science.

[fsn371018-bib-0027] He, J. , X. Wei , S. Li , et al. 2019. “DT‐13 Suppresses Breast Cancer Metastasis by Modulating PLOD2 in the Adipocytes Microenvironment.” Phytomedicine: International Journal of Phytotherapy and Phytopharmacology 59: 152778. 10.1016/j.phymed.2018.12.001. 31005809

[fsn371018-bib-0028] Hou, N. , and X. Sun . 2019. “Effect of Chinese Wolfberry Extract on Osteoporosis Rats and Its Mechanism.” Modern Food Science and Technology 35, no. 11: 37–44+108. 10.13982/j.mfst.1673-9078.2019.11.006.

[fsn371018-bib-0030] Hu, T. , S. Liu , M. Shen , W. Zhong , and X. Bao . 2021. “The Status and Development Trend of Chinese Wolfberry Product as Medicine and Food.” Journal of Xinyang Agriculture and Forestry University 31, no. 4: 105–109. 10.16593/j.cnki.41-1433/s.2021.04.033.

[fsn371018-bib-0031] Huang, K. , W. Dong , W. Liu , et al. 2019. “2‐O‐β‐d‐Glucopyranosyl‐l‐Ascorbic Acid, an Ascorbic Acid Derivative Isolated From the Fruits of *Lycium Barbarum* L., Modulates Gut Microbiota and Palliates Colitis in Dextran Sodium Sulfate‐Induced Colitis in Mice.” Journal of Agricultural and Food Chemistry 67, no. 41: 11408–11419. 10.1021/acs.jafc.9b04411. 31556290

[fsn371018-bib-0032] Huang, X. , Z. Liu , and Y. Song . 2018. “Research Progress on Cortex Lycii Radices in the Treatment of Diabetes Mellitus.” Guangming Journal of Chinese Medicine 33, no. 22: 3315–3317.

[fsn371018-bib-0033] Huang, Y. , Y. Zhang , F. Yang , et al. 2022. “ *Lycium barbarum* Glycopeptide Prevents the Development and Progression of Acute Colitis by Regulating the Composition and Diversity of the Gut Microbiota in Mice.” Frontiers in Cellular and Infection Microbiology 9, no. 12: 921075. 10.3389/fcimb.2022.921075. PMC9395742 36017369

[fsn371018-bib-0034] Inbaraj, B. S. , H. Lu , C. F. Hung , W. B. Wu , C. L. Lin , and B. H. Chen . 2008. “Determination of Carotenoids and Their Esters in Fruits of *Lycium barbarum* Linnaeus by HPLC‐DAD‐APCI‐MS.” Journal of Pharmaceutical and Biomedical Analysis 47, no. 4–5: 812–818. 10.1016/j.jpba.2008.04.001. 18486400

[fsn371018-bib-0035] Inbaraj, B. S. , H. Lu , T. H. Kao , and B. H. Chen . 2010. “Simultaneous Determination of Phenolic Acids and Flavonoids in *Lycium barbarum* Linnaeus by HPLC‐DAD‐ESI‐MS.” Journal of Pharmaceutical and Biomedical Analysis 51, no. 3: 549–556. 10.1016/j.jpba.2009.09.006. 19819093

[fsn371018-bib-0036] Ji, D. , D. Zhang , Z. Wang , G. Fan , and Q. Yu . 2022. “Characteristics of Nutrients in the Fruits of *Lycium Barbarum* Under Organic Cultivation.” Journal of Qinghai University 40, no. 5: 41–46. 10.13901/j.cnki.qhwxxbzk.2022.05.006.

[fsn371018-bib-0037] Jia, M. , P. Wang , M. Zhang , et al. 2014. “Effects of *Lycium barbarum* Leaf of Ningxia Transformed Products on Immune Function of Mice.” Journal of Liaoning University of TCM 16, no. 3: 5–7. 10.13194/j.issn.1673-842x.2014.03.001.

[fsn371018-bib-0038] Jiang, M. , and Z. Li . 2022. “ *Lycium Barbarum* Polysaccharide Induced Apoptosis of Human Tongue Squamous Cancer CAL‐27 Cells via the p53 Pathway.” Journal of Clinical Stomatology 38, no. 3: 140–143.

[fsn371018-bib-0039] Jiang, Y. , L. Yu , and M. Wang . 2015. “N‐Trans‐Feruloyltyramine Inhibits LPS‐Induced NO and PGE2 Production in RAW 264.7 Macrophages: Involvement of AP‐1 and MAP Kinase Signalling Pathways.” Chemico‐Biological Interactions 235: 56–62. 10.1016/j.cbi.2015.03.029. 25843058

[fsn371018-bib-0040] Kadiliya, M. , A. Ailigen , and J. Zhang . 2020. “A Regulating VEGF Signal Pathway in Rats With Otitis Media With Effusion.” Practical Pharmacy and Clinical Remedies 23, no. 12: 1069–1073. 10.14053/j.cnki.ppcr.202012003.

[fsn371018-bib-0041] Kim, J. , E. Kim , B. Lee , et al. 2016. “The Effects of *Lycii Radicis Cortex* on RANKL‐Induced Osteoclast Differentiation and Activation in RAW 264.7 Cells.” International Journal of Molecular Medicine 37, no. 3: 649–658. 10.3892/ijmm.2016.2477. 26848104 PMC4771095

[fsn371018-bib-0042] Lai, W. , C. Wang , R. Lai , X. Peng , and J. Luo . 2022. “ *Lycium Barbarum* Polysaccharide Modulates Gut Microbiota to Alleviate Rheumatoid Arthritis in a Rat Model.” NPJ Science of Food 6, no. 1: 34. 10.1038/s41538-022-00149-z. 35864275 PMC9304368

[fsn371018-bib-0043] Lei, L. 2014. Effects of Total Flavonoids in Lycium Chinensis Leaves on Proliferation and Apoptosis of Hepatoma Cell Line HepG2. Lanzhou University.

[fsn371018-bib-0044] Li, H. , M. Chen , W. Ma , and J. Wang . 2006. “Law of Changes of Carotenoids Contents in Fructus Lycii of Chinese Wolfberry (*Lycium Barbarum L*.) at Different Mature Periods.” Scientia Agricultura Sinica 3: 599–605.

[fsn371018-bib-0045] Li, M. , B. Cui , and Z. Zhang . 2024. “Comparative Analysis of Main Components and In Vitro Antioxidant Activities of *Lycium Barbarum* Fruits From Different Producing Areas.” Food Research and Development 45, no. 12: 7–12.

[fsn371018-bib-0046] Li, M. , Y. Zhang , C. Liu , C. Niu , Q. Li , and J. Wang . 2021. “Optimization of Fermentation Process and Analysis of Aroma Components in Wolfberry Wine.” Journal of Anhui Agricultural University 8, no. 5: 865–872. 10.13610/j.cnki.1672-352x.20211022.003.

[fsn371018-bib-0047] Li, X. , J. Lin , B. Chen , H. Xie , and D. Chen . 2018. “Antioxidant and Cytoprotective Effects of Kukoamines A and B: Comparison and Positional Isomeric Effect.” Molecules 23, no. 4: 973. 10.3390/molecules23040973. 29690528 PMC6017596

[fsn371018-bib-0048] Li, X. 2023. Study on Dynamic Accumulation of Chemical Constituents and Antioxidant and Hypoglycemic Activity in Lycii Cortex. Hebei University of Chinese Medicine. 10.27982/d.cnki.ghbyz.2021.000067.

[fsn371018-bib-0049] Li, Y. , Q. Li , Y. Guo , Z. Hao , and S. Jin . 2024. “Establishment of Sensory Evaluation Method for Wolfberry Tea and Exploration of the Main Characteristic Aroma Substances.” Science and Technology of Food Industry 46, no. 4: 1–22. 10.13386/j.issn1002-0306.2024020180.

[fsn371018-bib-0050] Li, Y. , L. Wang , and Y. Zhou . 2003. “Experimental Study on the Immune Enhancement and Anti Lipid Peroxidation Effects of *Lycium barbarum* Polysaccharides.” Acta Chinese Medicine and Pharmacology 2: 25. 10.19664/j.cnki.1002-2392.2003.02.017.

[fsn371018-bib-0051] Li, Z. , H. Liu , and F. Zhou . 2011. “Pharmacodynamic Study on the Treatment of Skin Burns in Mice With Cortex Lycii.” Journal of Chinese Medicinal Materials 34, no. 8: 1266–1270. 10.13863/j.issn1001-4454.2011.08.034.

[fsn371018-bib-0052] Li, Z. , J. Zhang , X. Ren , Q. Liu , and X. Yang . 2018. “The Mechanism of Quercetin in Regulating Osteoclast Activation and the PAR2/TRPV1 Signaling Pathway in the Treatment of Bone Cancer Pain.” International Journal of Clinical and Experimental Pathology 11, no. 11: 5149–5156. 31949595 PMC6963045

[fsn371018-bib-0053] Liang, N. , L. Du , Y. Ma , et al. 2023. “The Effect of *Lycium Barbarum* Polysaccharides on NLRP3 Inflammasome/Pyroptosis Pathway in High Glucose‐Induced Human Retinal Pigment Epithelial Cells.” Journal of Ningxia Medical University 45, no. 7: 649–654. 10.16050/j.cnki.issn1674-6309.2023.07.001.

[fsn371018-bib-0054] Liang, X. , Y. Xin , X. Jia , X. Yan , Y. Wei , and H. Zhao . 2021. “Response of Antioxidative Enzymes Activities and Amino Acids Concentrations in Leaf Tissues of *Lycium Barbarum L*. Seedlings Under Cadmium Stress.” Asian Journal of Ecotoxicology 16, no. 6: 222–233.

[fsn371018-bib-0055] Liao, J. , Q. Cai , and X. Zhang . 2023. “Effects of IL‐33/ST2 Signal on Immune Function and Sodium Channel in Otitis Media Rats by Using Tiosteopantin.” Chinese Pharmacological Bulletin 39, no. 10: 1853–1858.

[fsn371018-bib-0056] Liao, J. , J. Guo , Y. Niu , T. Fang , F. Wang , and Y. Fan . 2022. “Flavonoids From *Lycium Barbarum* Leaves Attenuate Obesity Through Modulating Glycolipid Levels, Oxidative Stress, and Gut Bacterial Composition in High‐Fat Diet‐Fed Mice.” Frontiers in Nutrition 9: 972794. 10.3389/fnut.2022.972794. 35967795 PMC9366397

[fsn371018-bib-0057] Liu, H. 2020. Study on the Anti‐Fatigue and Anti‐Diabetes Effects of Lycium barbarum L. Water Extracts by Regulating Oxidative Stress Pathway. Jilin University. 10.27162/d.cnki.gjlin.2019.000669.

[fsn371018-bib-0058] Liu, H. , Z. Zang , J. Li , et al. 2022. “Oligosaccharides Derived From *Lycium Barbarum* Ameliorate Glycolipid Metabolism and Modulate the Gut Microbiota Community and the Faecal Metabolites in a Type 2 Diabetes Mouse Model: Metabolomic Bioinformatic Analysis.” Food & Function 13, no. 9: 5416–5429. 10.1039/d1fo02667d. 35475434

[fsn371018-bib-0059] Liu, Q. , Y. Li , S. Sheng , and Y. Li . 2020. “Effects of *Lycium Barbarum* Polysaccharide on Learning and Memory of Aging Mice.” Journal of Lanzhou University (Medical Sciences) 46, no. 3: 38–42. 10.13885/j.issn.1000-2812.2020.03.007.

[fsn371018-bib-0060] Liu, S. , M. Yang , Y. Li , et al. 2019. “Variance Analysis on Polysaccharide, Total Flavonoids and Total Phenols of *Lycium barbarum* Leaves From Different Production Areas.” China Journal of Chinese Materia Medica 44, no. 9: 1774–1780. 10.19540/j.cnki.cjcmm.20190325. 31342701

[fsn371018-bib-0061] Liu, T. , Y. Yang , T. Lei , Y. Zhang , and L. Ming . 2016. “Acute Toxicity of Ethyl Acetate Extraction From Fructus Lycii and Its Effect on Osteoporosis in Ovariectomized Rats.” Chinese Journal of Osteoporosis 22, no. 4: 396–401.

[fsn371018-bib-0062] Liu, W. , Q. Song , Y. Zu , et al. 2020. “Neuroprotective Effects of Kukoamine a Against Rotenone‐Induced Neurotoxicity in PC12 Cells.” Chinese Traditional and Herbal Drugs 51, no. 24: 6302–6309.

[fsn371018-bib-0066] Liu, Y. 2016. Study on Extraction, Purification, Characterization and Biological Activity of Polysaccharides From Lycium Ruthenicum Leaves. Northwestern University.

[fsn371018-bib-0063] Liu, Y. , Y. Jin , F. Meng , and H. Yu . 2016. “Anti‐Fatigue Activity of *Lycium barbarum* Polysaccharides in Mice by Attenuating Oxidative Stress.” Science and Technology of Food Industry 37, no. 18: 344–348. 10.13386/j.issn1002-0306.2016.18.057.

[fsn371018-bib-0064] Liu, Y. , J. Liu , Z. Kong , et al. 2022. “Transcriptomics and Metabolomics Analyses of the Mechanism of Flavonoid Synthesis in Seeds of Differently Colored Quinoa Strains.” Genomics 114, no. 1: 138–148. 10.1016/j.ygeno.2021.11.030. 34863898

[fsn371018-bib-0065] Liu, Y. , M. Long , X. Tian , et al. 2019. “Extraction Process Optimization and Determination of Total Flavonoids in Chinese Wolfberry in Different Years.” Journal of Northwest Minzu University (Natural Science Edition) 40, no. 3: 70–75. 10.14084/j.cnki.cn62-1188/n.2019.03.013.

[fsn371018-bib-0067] Lu, L. , X. Lu , M. Yuan , Z. Li , S. Mei , and X. Li . 2021. “Research Progress on Chemical Constituents of Lycii Cortex.” Yunnan Chemical Technology 48, no. 8: 8–14.

[fsn371018-bib-0068] Lu, L. , J. Mi , Q. Luo , Y. Li , and X. Liang . 2019. “Optimization of Extraction Process of Flavonoids From *Lycium Barbarum L*. Var. *Auranticarpum K.F*. Ching and Its Antioxidant Activities In Vitro.” Science and Technology of Food Industry 40, no. 24: 165–171. 10.13386/j.issn1002-0306.2019.24.027.

[fsn371018-bib-0069] Lu, Y. , S. Guo , F. Zhang , et al. 2019. “Systematic Utilization and Industrialization Development of Medicinal Plant Resources of *Lycium Genus* .” Modern Chinese Medicine 21, no. 1: 9–36. 10.13313/j.issn.1673-4890.20181225001.

[fsn371018-bib-0070] Luo, Q. , L. Lu , Y. Yan , et al. 2022. “Immunomodulatory Effects of Spray Dried Powder of Goji (*Lycium Barbarum L*.) and Goji Polysaccharides on Immunosuppressive Mice Induced by Cyclophosphamide and Their Regulation on Gut Microbiota.” Food Science 43: 137–148.

[fsn371018-bib-0071] Luo, Y. , H. Fu , J. Suo‐Lang , and Y. Zhou . 2024. “Preparation and Analyses of Textural Quality and Flavor Substances of Goji Berry Yoghurt.” Modern Food Science and Technology 40, no. 1: 249–261.

[fsn371018-bib-0072] Lv, M. J. 2021. Comprehensive Evaluation of the Main Chemical Components and Biological Activities of Goji Leaves and Study on Their High‐Efficient Extraction and Enrichment Technology. Beijing Forestry University. 10.26949/d.cnki.gblyu.2021.000572.

[fsn371018-bib-0073] Ma, F. , J. Ma , F. Gong , and J. Gao . 2016. “Effects of Leaves of *Lycium Barbarum* on Estrogen Receptor in Ovariectomized Rats.” Chinese Journal of Tissue Engineering Research 15, no. 20: 2178–2183. 10.3969/j.issn.2095-4344.2016.15.007.

[fsn371018-bib-0074] Ma, M. 2024a. Comprehensive Extraction of Active Substances From Lycium Barbarum L. Leaves and Screening of Their Biological Activities In Vitro. Ningxia University. 10.27257/d.cnki.gnxhc.2023.001978.

[fsn371018-bib-0075] Ma, Q. , J. Zhang , and X. Ran . 2000. “The Effects of Compound Goji Berry Leaf Tea on Fatigue Resistance, Hypoxia Tolerance, Low Temperature Tolerance, and Blood Lipids.” Journal of Xinjiang Medical University 2: 153–154.

[fsn371018-bib-0076] Ma, Q. , L. Zhang , L. Lu , et al. 2024. “Progress in Research and Application of Phenols and Their Derivatives From *Lycium Barbarum L* .” Food Science 45, no. 17: 316–325.

[fsn371018-bib-0077] Ma, R. 2024b. Study on the Difference of Biosynthesis of Flavonoids in ‘Ningqi No.1’ *Lycium Barbarum* From Different Regions Based on Multiomics Technology. Ningxia University. 10.27257/d.cnki.gnxhc.2023.001815.

[fsn371018-bib-0078] Mei, L. 2016. “Research and Industrialization of Chaidamu Goji Leaf Flavonoids Compound Soft Capsules.”

[fsn371018-bib-0079] Mohammed, Z. , K. Alsamarrae , and S. Hamza . 2024. “Preliminary Study for the Anticancer Activity of Flavonoids Extracted From Wild *Lycium Barbarum* Leaves.” International Journal of Research Studies in Biosciences 3: 88–94. http://arcjournals.org/pdfs/ijrsb/v3‐i12/12.pdf.

[fsn371018-bib-0080] Mu, B. , X. Liu , J. Xiong , and I. Gou . 2002. “Acute Toxicity and Mutagenicity of *Lycium barbarum* Polysaccharides.” Journal of Environmental and Occupational Medicine 3: 201–202. 10.13213/j.cnki.jeom.2002.03.089.

[fsn371018-bib-0082] Niu, Y. , J. Liao , H. Zhou , C. C. Wang , L. Wang , and Y. Fan . 2022. “Flavonoids From *Lycium Barbarum* Leaves Exhibit Anti‐Aging Effects Through the Redox‐Modulation.” Molecules (Basel, Switzerland) 27, no. 15: 4952. 10.3390/molecules27154952. 35956901 PMC9370597

[fsn371018-bib-0083] Oh, Y. C. , W. K. Cho , G. Y. Im , et al. 2012. “Anti‐Inflammatory Effect of Lycium Fruit Water Extract in Lipopolysaccharide‐Stimulated RAW 264.7 Macrophage Cells.” International Immunopharmacology 13, no. 2: 181–189. 10.1016/j.intimp.2012.03.020. 22483979

[fsn371018-bib-0084] Olatunji, O. , H. Chen , and Y. Zhou . 2017. “Effect of the Polyphenol Rich Ethyl Acetate Fraction From the Leaves of *Lycium Chinensemill*. On Oxidative Stress, Dyslipidemia, and Diabetes Mellitus in Streptozotocin‐Nicotinamide Induced Diabetic Rats.” Chemistry & Biodiversity 14, no. 10: e1700277. 10.1002/cbdv.201700277. 28677319

[fsn371018-bib-0085] Paek, E. , H. Jin , D. Cho , et al. 2014. “The Effect of *Lycii Radicis Cortex* Extract on Bone Formation In Vitro and In Vivo.” Molecules (Basel, Switzerland) 19, no. 12: 19594–19609. 10.3390/molecules191219594. 25432011 PMC6271141

[fsn371018-bib-0086] Pan, C. , C. Zhang , Z. Lin , et al. 2024. “Disulfidptosis‐Related Protein RPN1 May Be a Novel Anti‐Osteoporosis Target of Kaempferol.” Combinatorial Chemistry & High Throughput Screening 27, no. 11: 1611–1628. 10.2174/0113862073273655231213070619. 38213143

[fsn371018-bib-0087] Pan, G. , L. Duan , J. Zhao , et al. 2024. “Effect of the Flavor and Quality of Wolfberry Tea Processed With Buds and Leaves Under Different Degree of Spreading.” Science and Technology of Food Industry 46, no. 4: 1–17. 10.13386/j.issn1002-0306.2024040128.

[fsn371018-bib-0088] Park, E. , J. Kim , S. Yeo , et al. 2019. “Anti‐Osteoporotic Effects of Combined Extract of *Lycii Radicis Cortex* and *Achyranthes Japonica* in Osteoblast and Osteoclast Cells and Ovariectomized Mice.” Nutrients 11, no. 11: 2716. 10.3390/nu11112716. 31717518 PMC6893723

[fsn371018-bib-0089] Peng, H. 2014. “Protective Effects of Lycii Cortex Ethanol Extract on Diabetic Model Rats.” China Pharmacy 25, no. 27: 2513–2515.

[fsn371018-bib-0090] Peng, M. , Y. Zhou , and B. Liu . 2024. “Biological Properties and Potential Application of Extracts and Compounds From Different Medicinal Parts (Bark, Leaf, Staminate Flower, and Seed) of *Eucommia Ulmoides*: A Review.” Heliyon 10, no. 6: e27870. 10.1016/j.heliyon.2024.e27870. 38545153 PMC10966601

[fsn371018-bib-0091] Pharmacology Teaching and Research Group . 1959. “Antihypertensive Effect of Cortex Lycii and Lotus Leaf.” Journal of Nanjing Medical University (Natural Sciences) 3: 255–259.

[fsn371018-bib-0092] Pollini, L. , A. Riccio , C. Juan , C. Tringaniello , F. Ianni , and F. Blasi . 2020. “Phenolic Acids From *Lycium Barbarum* Leaves: In Vitro and In Silico Studies of the Inhibitory Activity Against Porcine Pancreatic α‐Amylase.” Processes 8, no. 11: 1388.

[fsn371018-bib-0093] Qin, S. , Q. Bian , G. Hou , et al. 1988. “Toxicity Evaluation of Northern Wolfberry.” Journal of Toxicology 3: 177. 10.16421/j.cnki.1002-3127.1988.03.019.

[fsn371018-bib-0094] Qingdao Hengbo Instrument Co., Ltd . 2014. A Lipid‐Lowering and Hypoglycemic Cortex Lycii Health Tea and Its Preparation Method. Qingdao Hengbo Instrument Co., Ltd.

[fsn371018-bib-0095] Qingdao Hengbo Instrument Co., Ltd . 2017. A Type of Health Tea Made From Cortex Lycii That Enhances Immunity and Its Preparation Method. Qingdao Hengbo Instrument Co., Ltd.

[fsn371018-bib-0096] Qu, F. , Y. Bian , Y. Yu , and Y. An . 2020. “ *Lycium barbarum* Polysaccharide Inhibits Inflammatory Response in Rats With Diabetic Nephropathy via TLR4/Myd88 Pathway.” Progress of Anatomical Sciences 26, no. 4: 378–382. 10.16695/j.cnki.1006-2947.2020.04.003.

[fsn371018-bib-0097] Qu, T. , Q. Pan , Y. Wen , and L. Zhang . 2020. “Acute Toxicity and Pharmacodynamics of *Lycium Barbarum* L. Combined With American Ginseng.” Hunan Journal of Traditional Chinese Medicine 36, no. 11: 186–188+203. 10.16808/j.cnki.issn1003-7705.2020.11.070.

[fsn371018-bib-0098] Quan, N. 2018. Isolation, Purification, Structural Characterization, and Hypoglycemic Activity Analysis of Polysaccharides From Goji Berry Leaves. Shanxi Normal University.

[fsn371018-bib-0099] Quan, N. , Y. D. Wang , G. R. Li , et al. 2023. “Ultrasound‐Microwave Combined Extraction of Novel Polysaccharide Fractions From *Lycium Barbarum* Leaves and Their In Vitro Hypoglycemic and Antioxidant Activities.” Molecules 28, no. 9: 3880. 10.3390/molecules28093880. 37175290 PMC10180117

[fsn371018-bib-0100] Shan, T. , Y. Liu , and S. Yang . 2010. “Inhibitory Effect of *Lycium Barbarum* Polysaccharide on Proliferation of Human Esophageal Carcinoma Cells and the Relationship With Rb Protein Expression.” Lishizhen Medicine and Materia Medica Research 21, no. 9: 2405–2406.

[fsn371018-bib-0101] Shan, Y. , T. Pang , Y. Yuan , and H. Chen . 2024. “Effect of *Digupi (Lycii Cortex)* on Intestinal Flora in Rats With Spontaneous Hypertension Based on 16S rDNA Sequencing.” Journal of Practical Traditional Chinese Internal Medicine 38, no. 4: 1–3+143‐144. 10.13729/j.issn.1671-7813.Z20232383.

[fsn371018-bib-0102] Shao, C. , Y. Deng , B. Yang , et al. 2020. “The Nutritional Value and Health Function of *Lycium Barbarum* and Its Application Progress.” Anhui Agricultural Science Bulletin 26, no. 17: 39–41.

[fsn371018-bib-0103] Song, Y. , T. Peng , S. Ma , et al. 2024. “Analyzing the Autotoxicity of Phenolic Acids From *Lycium Barbarum L*. Leaves.” Jiangsu Journal of Agricultural Sciences 40, no. 2: 213–222.

[fsn371018-bib-0104] Su, Y. , J. Xi , S. Dong , S. Shao , Q. Liang , and F. Yang . 2024. “Research Progress on Extraction, Separation, Purification, and Pharmacological Activities of Pigments Extracted From *Lycium Barbarum* .” Chinese Archives of Traditional Chinese Medicine 43, no. 2: 1–10.

[fsn371018-bib-0105] Sun, C. , X. Chen , S. Yang , C. Jin , K. Ding , and C. Chen . 2023. “LBP1C‐2 From *Lycium Barbarum* Alleviated Age‐Related Bone Loss by Targeting BMPRIA/BMPRII/Noggin.” Carbohydrate Polymers 310: 120725. 10.1016/j.carbpol.2023.120725. 36925250

[fsn371018-bib-0106] Sun, J. , Y. Liu , P. Cong , X. Shi , R. Ma , and H. Jin . 2022. “Effect of Kukoamine B on Oxidative Stress Injury of Lung During Low Temperature Detonation.” Clinical Journal of Medical Officers 50, no. 3: 233–236. 10.16680/j.1671-3826.2022.03.04.

[fsn371018-bib-0107] Sun, J. , C. Wu , and J. Fan . 2020. “On the Development Status of and Measuresfor Fruit Wine Industry in China.” Liquor‐Making Science & Technology 12: 124–127. 10.13746/j.njkj.2020129.

[fsn371018-bib-0108] Taskan, M. , and F. Gevrek . 2020. “Quercetin Decreased Alveolar Bone Loss and Apoptosis in Experimentally Induced Periodontitis Model in Wistar Rats.” Anti‐Inflammatory & Anti‐Allergy Agents in Medicinal Chemistry 19, no. 4: 436–448. 10.2174/1871523019666200124114503. 31976849

[fsn371018-bib-0109] Tong, J. , Y. Chen , X. Li , et al. 2024. “Researches on Effective Fraction and Mechanism of *Lycium Barbarum* Leaves on Improving Learning and Memory Abilities of D‐Galactose‐Induced Subacute Aging Mice.” Modernization of Traditional Chinese Medicine and Materia Medica‐World Science and Technology 26, no. 1: 48–60.

[fsn371018-bib-0110] Wang, C. , M. Yue , D. Wang , and J. Wang . 2015. “Preliminary Study on Antidepressant Effect of Total Flavonoids of *Lycium Barbarum* Leaves.” Journal of Ningxia University (Natural Science Edition) 36, no. 3: 261–266.

[fsn371018-bib-0111] Wang, H. , X. Bai , and P. Bao . 2006. “The Effects of *Lycium Barbarum* on the Immunopharmacdogical Activity of the Mice.” Jinzhou Medical University 1: 47–49.

[fsn371018-bib-0112] Wang, J. , M. Ren , Y. Wang , et al. 2015. “Effects of *Lycium barbarum* Leaves and *Lycium Larbarum* Polysaccharides on Osteoporosis in Ovariectomized Rats.” Journal of Ningxia Medical University 37, no. 3: 233–236+227. 10.16050/j.cnki.issn1674-6309.2015.03.001.

[fsn371018-bib-0113] Wang, J. , B. Zhang , F. Zhou , M. Wang , and J. Fan . 2020. “Nutritional Composition Analysis on *Lycium Barbarum* Leaves in Different Grow Ages.” Food and Nutrition in China 26, no. 12: 56–58+31. 10.19870/j.cnki.11-3716/ts.20201106.001.

[fsn371018-bib-0114] Wang, L. , W. Hu , H. Li , Y. Xu , and H. Liu . 2024. “Chemical Constituents of Methanol Fraction From Lycii Cortex and Their Anti‐Inflammatory Activities.” Chinese Traditional Patent Medicine 46, no. 5: 1540–1545.

[fsn371018-bib-0115] Wang, Q. , H. Li , Z. Sun , et al. 2016. “Kukoamine A Inhibits Human Glioblastoma Cell Growth and Migration Through Apoptosis Induction and Epithelial‐Mesenchymal Transition Attenuation.” Scientific Reports 6: 36543. 10.1038/srep36543. 27824118 PMC5099904

[fsn371018-bib-0116] Wang, Q. , G. Xu , Z. Zhang , S. Chen , and B. Yu . 1993. “Effects of *Lycium Barbarum* and *Lycii Cortex* Polysaccharides on Immune System in Mice.” Pharmacology and Clinics of Chinese Materia Medica 3: 39–40.

[fsn371018-bib-0117] Wang, Y. , J. Shan , Y. Chen , C. Qiao , and X. Luo . 2020. “Optimization of Fermentation Technology and Analysis of Flavor Substances of Wolfberry.” Food Research and Development 41, no. 18: 40–47.

[fsn371018-bib-0118] Wang, Z. , X. Chen , and J. Zhang . 2018. “Effects of *Lycium Barbarum* Polysaccharide Combined With Cisplatinon Inhibitory and Immune Function of Hepatic Cancer Mice.” Journal of Southeast University (Medical Science Edition) 37, no. 5: 896–900.

[fsn371018-bib-0119] Wei, X. , S. Lin , Y. Liu , et al. 2016. “DT‐13 Attenuates Human Lung Cancer Metastasis via Regulating NMIIA Activity Under Hypoxia Condition.” Oncology Reports 36, no. 2: 991–999. 10.3892/or.2016.4879. 27374701

[fsn371018-bib-0120] Wei, X. , H. Wang , Z. Sun , X. Sun , and H. Zhou . 2018. “Research Progress on Chemical Components and Pharmacological Activities of Ningxia Wolfberry.” Chinese Traditional Patent Medicine 40, no. 11: 2513–2520.

[fsn371018-bib-0121] Wei, Z. , J. Yang , Y. Tan , and J. Wang . 2012. “Study on the Hypoglycemic Effect of Ningxia Goji Berry Leaves at Different Harvesting Periods.” Lishizhen Medicine and Materia Medica Research 23, no. 11: 2786–2787.

[fsn371018-bib-0122] Wei, Z. , H. Yu , and R. Fan . 2009. “The Effective Components From Cortex Lycii for Reduction of Blood Sugar.” Lishizhen Medicine and Materia Medica Research 20, no. 4: 848–850.

[fsn371018-bib-0123] Wu, Q. , Z. Liu , B. Li , Y. Liu , and P. Wang . 2024. “Immunoregulation in Cancer‐Associated Cachexia.” Journal of Advanced Research 58: 45–62. 10.1016/j.jare.2023.04.018. 37150253 PMC10982873

[fsn371018-bib-0124] Wu, X. 2022. Study of Flavonoids in *Lycium barbarum* Leaves on Changes in Meat Quality Associated With Broiler Chickens Induced by Short‐Duration Transport Stress. Ningxia University. 10.27257/d.cnki.gnxhc.2022.001991.

[fsn371018-bib-0125] Xie, L. , A. Atanasov , D. Guo , et al. 2014. “Activity‐Guided Isolation of NF‐κB Inhibitors and PPARγ Agonists From the Root Bark of * Lycium chinense Miller* .” Journal of Ethnopharmacology 152, no. 3: 470–477. 10.1016/j.jep.2014.01.029. 24512737

[fsn371018-bib-0126] Xiong, L. 2021. Research on Anti‐Aging Activity of the Goji Berry Extracts and Mechanism of Action. South China University of Technology.

[fsn371018-bib-0127] Xiong, X. , and W. Li . 1991. “Dual Directional Regulatory Effect of Partially Tonifying Solid Traditional Chinese Medicine on IL‐2 Production in Mouse Spleen Cells.” Chinese Journal of Experimental and Clinical Immunology 3, no. 4: 4.

[fsn371018-bib-0128] Xu, H. , L. Zhao , X. Zhang , J. Hua , M. Wang , and H. Zhao . 2021. “Research Progress on the Chemical Constituents and Biological Effects of Cortex Lycii.” China Surfactant Detergent & Cosmetics 51, no. 5: 450–456+467.

[fsn371018-bib-0129] Yan, H. , J. F. Xue , and L. Li . 2012. “Determination of Active Ingredient Content and Immune Performance in *Lycium Barbarum* Production and Processing Waste.” Feed Industry 33, no. 11: 57–59.

[fsn371018-bib-0130] Yan, Y. , Y. Ge , X. Zhang , et al. 2024. “Evaluating the Differences in Different Parts of *Lycium Barbarum L*. by Fingerprint‐Based Chemical Pattern Recognition Method and Multi‐Index Determination.” Journal of Hunan University of Chinese Medicine 44, no. 7: 1193–1202.

[fsn371018-bib-0131] Yan, Y. , L. Ran , Y. Cao , K. Qin , and X. Zeng . 2014. “Nutritional, Phytochemical Characterization and Antioxidant Capacity of Ningxia Wolfberry (*Lycium Barbarum L*.).” Journal of the Chemical Society of Pakistan 36, no. 6: 1079–1087.

[fsn371018-bib-0132] Yan, Z. , and T. Liu . 2011. “Effect of Water Extract of Cortex Rehmanniae on Glucose and Lipid Metabolism in Insulin Resistant Rats With Type 2 Diabetes Mellitus.” Yunnan Journal of Traditional Chinese Medicine and Materia Medica 32, no. 8: 56–58. 10.16254/j.cnki.53-1120/r.2011.08.037.

[fsn371018-bib-0133] Yang, L. , and Z. Ye . 2008. “Effect and Mechanisms of Cortex Lycii to Renal Lesions in T2DM Rats.” Chinese Archives of Traditional Chinese Medicine 10: 2172–2175. 10.13193/j.archtcm.2008.10.93.yangl.056.

[fsn371018-bib-0134] Yao, H. , J. Chen , S. Chen , and D. Zhou . 2020. “Experimental Study on the Improvement of Insulin Resistance in Rats With Type 2 Diabetes Mellitus With Cortex Lycii Radicis Based on AMPK/GLUT4/GSK3β/PPARα Signaling Pathway.” China Medical Herald 17, no. 5: 8–12+198.

[fsn371018-bib-0135] Yi, R. , X. M. Liu , and Q. Dong . 2013. “A Study of *Lycium barbarum* Polysaccharides (LBP) Extraction Technology and Its Anti‐Aging Effect.” African Journal of Traditional, Complementary, and Alternative Medicines: AJTCAM 10, no. 4: 171–174. 10.4314/ajtcam.v10i4.27. 24146519 PMC3794409

[fsn371018-bib-0137] Yin, F. , Z. Liu , X. Tian , Y. Wang , and T. Zhou . 2024. “Optimization of Purification Process and Study on Composition and Antioxidant Activity of Polyphenols From Goji Berry.” Chinese Traditional Patent Medicine 46, no. 9: 3078–3083.

[fsn371018-bib-0136] Yin, H. 2021. Study on Anti‐Lung Cancer Effect and Mechanism of Extracts of Lycii Radicis Cortex. Guangzhou University of Chinese Medicine. 10.27044/d.cnki.ggzzu.2021.000533.

[fsn371018-bib-0138] Yin, J. , D. Wang , F. Li , Y. Chen , and Z. Jiang . 2001. “The Effect of the Extracts of Some Crude Drugs on Proliferation of Osteoblast‐Like UMR106cells.” Journal of Shenyang Pharmaceutical University 4, no. 18: 279–282.

[fsn371018-bib-0139] Yu, C. , Y. Chen , S. Ahmadi , et al. 2023. “Goji Berry Leaf Exerts a Comparable Effect Against Colitis and Microbiota Dysbiosis to Its Fruit in Dextran‐Sulfate‐Sodium‐Treated Mice.” Food & Function 14, no. 7: 3026–3037. 10.1039/d2fo02886g. 36861301

[fsn371018-bib-0140] Yu, M. , and Z. Ye . 2012. “Effect of Digupi Extracts on the Expression of PPARγ on 3T3‐L1 Fat Cells Induced by Lipopolysaccha‐Ride.” Zhejiang JITCWM 22, no. 7: 517–518+575.

[fsn371018-bib-0141] Zang, H. , X. Liu , Y. Sun , and C. Yang . 2021. “Extraction of Polyphenols From Lycii Cortex Based on Aqueous Two‐Phase System and Evaluation of Its Antioxidant Capacity.” Applied Chemical Industry 50, no. 4: 855–859+867. 10.16581/j.cnki.issn1671-3206.2021.04.001.

[fsn371018-bib-0142] Zeng, H. , C. Yang , C. Jin , and K. Ding . 2024. “Exploration of the Active Domain of Polysaccharide LBP1C‐2 Targeting β‐Subunit‐2 of Voltage‐Gated Potassium Channel.” Modernization of Traditional Chinese Medicine and Materia Medica—World Science and Technology 26, no. 5: 1182–1191.

[fsn371018-bib-0143] Zeng, W. 2022. Structural Characterization, Digestive Characteristics and Biological Activities of Polysaccharides From *Lycium barbarum* . North Minzu University. 10.27754/d.cnki.gbfmz.2022.000333.

[fsn371018-bib-0144] Zhang, C. , L. Xie , and W. Sun . 2024. “Effect of *Lycium Barbarum* Polysaccharide on Insulin Resistance in Pregnant Diabetes Rats by Regulating Nrf2/HO‐1/GPX4 Iron Death Pathway.” Chinese Traditional Patent Medicine 46, no. 2: 626–630.

[fsn371018-bib-0145] Zhang, D. , X. Zhang , X. Hao , W. Li , B. Sun , and X. Zhou . 2014. “Effects of Total Flavonoids of *Lycium Barbarum L*.on Proliferation and Apoptosis of Human Gastric Cancer Cell Lines SGC‐7901.” Lishizhen Medicine and Materia Medica Research 25, no. 11: 2634–2637.

[fsn371018-bib-0146] Zhang, H. , and P. Liang . 2008. “Fructus Lycii Influence Investigation to Thymus Function in D‐Galactose In‐Duced Immunosenescence Mouse Model.” China Medical Herald 21: 13–14.

[fsn371018-bib-0147] Zhang, L. , B. Zhou , G. Xiao , et al. 2022. “Comparative Analysis of *Lycium Barbarum* Pulp Sterilization Using Combinations of Low‐Oxygen Beating and Other Techniques.” Modern Food Science and Technology 38, no. 11: 158–165. 10.13982/j.mfst.1673-9078.2022.11.0035.

[fsn371018-bib-0148] Zhang, Q. , X. Zhu , J. Xiong , and X. Luo . 2024. “Research Progress of Functional Properties of Wolfberry and Development of Its Products.” Food and Fermentation Industries 50, no. 15: 398–408. 10.13995/j.cnki.11-1802/ts.037637.

[fsn371018-bib-0149] Zhang, S. , C. Lu , H. Zheng , et al. 2024. “The Intervention Effect of *Lycium Barbarum* Leaves on Letrozole‐Induced PCOS Mice Based on Microbiome.” Acta Pharmaceutica Sinica 59, no. 7: 2030–2040. 10.16438/j.0513-4870.2023-1449.

[fsn371018-bib-0150] Zhang, W. , Y. Lin , H. Liu , and W. Zhang . 2022. “Study on the Hypoglycemic Activity of Flavonoids From *Lycium barbarum* . *Heilongjiang* .” Science 13, no. 16: 1–3+18.

[fsn371018-bib-0151] Zhang, X. , Y. Dai , Y. Wang , et al. 2024. “Research Progress of Gouqi (*Lycium Barbarum*) and Predictive Analysis on Its Q‐Markers.” Chinese Archives of Traditional Chinese Medicine 42: 174–183+260. 10.13193/j.issn.1673-7717.2024.01.032.

[fsn371018-bib-0152] Zhao, J. , J. Xiao , C. Shi , L. Wang , B. He , and X. Gao . 2020. “Optimization of Extraction Process of *Lycium Barbarum* Leaf Polysaccharide and Its Alleviation of Allergic Rhinitis in Mice.” Food and Fermentation Industries 46, no. 24: 90–96. 10.13995/j.cnki.11-1802/ts.025203.

[fsn371018-bib-0153] Zhao, R. , T. Zhang , Y. Cai , et al. 2015. “The Effect of *Lycium Barbarum* Polysaccharide Capsules on Acute Toxicity in Mice.” Chinese Journal of Gerontology 35, no. 20: 5714–5716.

[fsn371018-bib-0154] Zhao, X. Q. , S. Guo , Y. Y. Lu , et al. 2020. “ *Lycium barbarum L*. Leaves Ameliorate Type 2 Diabetes in Rats by Modulating Metabolic Profiles and Gut Microbiota Composition.” Biomedicine & Pharmacotherapy = Biomedecine & Pharmacotherapie 121: 109559. 10.1016/j.biopha.2019.109559. 31734581

[fsn371018-bib-0155] Zhao, X. Q. 2020. Study on Resource Chemistry of Lycium Barbarum Leaves and Flowers. Nanjing University of Traditional Chinese Medicine. 10.27253/d.cnki.gnjzu.2020.000088.

[fsn371018-bib-0156] Zheng, H. , Y. Hua , X. Liu , et al. 2023. “Screening of Active Components and Preliminary Mechanism Exploration of Lycii Fructus for Improving Osteoporosis Based on the Zebrafish Model.” Acta Pharmaceutica Sinica 58, no. 1: 127–138. 10.16438/j.0513-4870.2022-200626.

[fsn371018-bib-0157] Zheng, H. L. , M. T. Li , T. Zhou , et al. 2023. “Protective Effects of *Lycium Barbarum L*. Berry Extracts Against Oxidative Stress‐Induced Damage of the Retina of Aging Mouse and ARPE‐19 Cells.” Food & Function 14, no. 1: 399–412. 10.1039/d2fo02788g. 36512065

[fsn371018-bib-0158] Zhou, J. , S. Guo , J. Li , H. Kang , L. Guo , and J. Duan . 2023. “Chemical Composition Analysis and Antioxidant Activity Evaluation of *Lycium Barbarum* Leaves With Different Strains and Maturity.” Journal of Nanjing University of Traditional Chinese Medicine 39, no. 9: 839–848. 10.14148/j.issn.1672-0482.2023.0839.

[fsn371018-bib-0159] Zhou, X. , L. Chan , B. Yi , et al. 2022. “Research on Active Components of *Lycium Chinense* and Their Pharmacological Effects.” Hubei Agricultural Sciences 61, no. 18: 120–130. 10.14088/j.cnki.issn.0439-8114.2022.18.021.

[fsn371018-bib-0160] Zhu, J. , W. Liu , J. Yu , et al. 2013. “Characterization and Hypoglycemic Effect of a Polysaccharide Extracted From the Fruit of * Lycium barbarum L* .” Carbohydrate Polymers 98, no. 1: 8–16. 10.1016/j.carbpol.2013.04.057. 23987311

[fsn371018-bib-0161] Zhu, Y. , T. Qian , X. Zhou , L. Cheng , and Z. Jin . 2022. “Application Status and Industrial Development Strategy of Goji Berry for Leaf Use. *Contemporary* .” Horticulture 45, no. 24: 37–38+42.

